# iPSC-Derived Embryoid Bodies as Models of c-*Met*-Mutated Hereditary Papillary Renal Cell Carcinoma

**DOI:** 10.3390/ijms20194867

**Published:** 2019-09-30

**Authors:** Jin Wook Hwang, Christophe Desterke, Olivier Féraud, Stephane Richard, Sophie Ferlicot, Virginie Verkarre, Jean Jacques Patard, Julien Loisel-Duwattez, Adlen Foudi, Frank Griscelli, Annelise Bennaceur-Griscelli, Ali G Turhan

**Affiliations:** 1 INSERM UMR-S 935 and ESTeam Paris Sud, Université Paris Sud, 94800 Villejuif, France; jinwook.hwang@inserm.fr (J.W.H.); christophe.desterke@inserm.fr (C.D.); olivier.feraud@inserm.fr (O.F.) ; adlen.foudi@inserm.fr (A.F.); frank.griscelli@gmail.com (F.G.); abenna@hotmail.fr (A.B.-G.); 2 Réseau National de Référence pour Cancers Rares de l’Adulte PREDIR, labellisé par l’INCa, et Service d’Urologie, AP-HP, Hôpital Bicêtre, 94270 Le Kremlin-Bicêtre, France; Génétique Oncologique EPHE, PSL Université, INSERM UMR 1186, Gustave Roussy, Faculté de Médecine et Université Paris-Sud, 94800 Villejuif, France; stephane.richard@u-psud.fr; 3 INSERM, UMR 1186, Gustave Roussy, Paris-Sud University, Paris-Saclay University, 94800 Villejuif, France; sophie.ferlicot@aphp.fr; 4 Department of Pathology, Bicêtre Hospital, AP-HP, 94270 Le Kremlin-Bicêtre, France; 5 Service d’Anatomie Pathologique, Hôpital Européen Georges Pompidou, AP-HP, 75015 Paris, France; virginie.verkarre@aphp.fr; 6 Faculté de médecine, Université Paris Descartes, 75006 Paris, France; 7 Service d’Urologie, Centre Hospitalier de Mont de Marsan, 40024 Mont de Marsan, France; jean-jacques.patard@ch-mdm.fr; 8 INSERM U1195, Université Paris Sud, Faculté de Médecine, APHP, Service de Neurologie, Hôpital Bicêtre, 94276 le Kremlin Bicêtre, France; julien.loisel-duwattez@inserm.fr; 9 ATIP Avenir INSERM UMR-S 935, Université Paris Sud, 94800 Villejuif, France; 10 INGESTEM National IPSC Infrastructure, 94800 Villejuif, France; 11 Paris Descartes University, Faculty Sorbonne Paris Cité, Faculté des Sciences Pharmaceutiques et Biologiques, 75006 Paris, France; 12 Division of Hematology, Paris Sud University Hospitals, Le Kremlin Bicêtre 75006, 94800 Villejuif, France

**Keywords:** Hereditary papillary renal cell carcinoma, c-*Met* mutation, induced pluripotent stem cell, kidney cancer organoids

## Abstract

Hereditary cancers with cancer-predisposing mutations represent unique models of human oncogenesis, as a driving oncogenic event is present in germline. Currently, there are no satisfactory models to study these malignancies. We report the generation of IPSC from the somatic cells of a patient with hereditary c-*met-*mutated papillary renal cell carcinoma (PRCC). From these cells we have generated spontaneous aggregates organizing in structures which expressed kidney markers such as PODXL and Six2. These structures expressed PRCC markers both in vitro and in vivo in NSG mice. Gene-expression profiling showed striking molecular similarities with signatures found in a large cohort of PRCC tumor samples. This analysis, applied to primary cancers with and without c-*met* mutation, showed overexpression of the BHLHE40 and KDM4C only in the c-*met*-mutated PRCC tumors, as predicted by c-*met*-mutated embryoid bodies transcriptome. These data therefore represent the first proof of concept of “hereditary renal cancer in a dish” model using c-*met*-mutated iPSC-derived embryoid bodies, opening new perspectives for discovery of novel predictive progression markers and for drug-screening for future precision-medicine strategies.

## 1. Introduction

Hereditary cancers are due to oncogenic mutations or deletions and represent a major challenge in terms of diagnosis, prognosis, and prevention [1]. Recently, the development of the iPSC technology allowed these cancers to be modeled using patient-derived pluripotent stem cells [2,3,4]. It has been shown that this strategy can be used for modeling BRCA1-mutated breast cancer [5] and Li–Fraumeni syndrome with TP53 deletions [3]. The major problem regarding the modeling of cancer using iPSC is that the effects of oncogenic mutations may appear only in the tissue in which the cancer develops [6]. Indeed, in a model of pancreatic cancer using iPSC, cancer phenotype appeared only when the specification of the endoderm had been achieved [6]. Similarly, the use of drug targeting strategies requires the generation of tissue-specific embryoid bodies, a technology which has been achieved in many tissues using either primary cells or pluripotent stem cells [7]. However, the feasibility and clinical use of iPSC-derived cancer embryoid bodies harboring a hereditary oncogenic mutation has not been shown so far. We show here the proof of concept of the feasibility of this strategy by generating iPSC lines bearing a c-*met* mutation. We developed from these patient-specific iPSC, embryoid bodies using a 3D in vitro culture system followed by extensive analysis by immunochemistry, transmission electron microscopy, and transcriptome studies. 

The results reported here show the possibility of generating structures reminiscent of kidney embryoid bodies with phenotypic PRCC markers using an in vitro, 3D culture system and in vivo after transplantation into NSG mice. Strikingly, transcriptome analysis of c-*met*-mutated-iPSC-derived renal progenitor cells revealed a gene profiling pattern similar to that reported in c-*met*-mutated primary human PRCC. In addition, the markers that we have discovered in iPSC-derived embryoid bodies are overexpressed only in primary PRCC with c-*met*-mutation. These results validate the feasibility of generating unlimited sources of embryoid bodies structures from iPSC for future drug-screening strategies in all hereditary cancers.

## 2. Results

### 2.1. Patients

The propositus patient is a patient in whom the diagnosis of the c-*met* mutation was performed during a genetic study realized in her family, because of the occurrence of PRCC in her mother. This analysis showed the presence of a mutation in the c-*met* oncogene atc.3900 G > A, p.Val1238Ile. After the molecular diagnosis, the patient had regular follow-ups in the urology clinic using nuclear magnetic resonance (NMR) imaging. In 2005, she developed two kidney carcinomas. Since this date, the stability of the disease allowed an annual radiological assessment and follow-up in urology clinic without specific therapy. 

The characteristics of 5 additional patients whose tumors have been analyzed in the study are shown in Table S1. The mother of the patient (UPN5) had developed two recurrent, c-*met*-mutated PRCC (Tumor Sample UPN5) and presented as expected, the same mutation as the patient (c.3900 G > A, p.Val1238Ile). A second patient with c-*met*-mutated PRCC (Tumor Sample UPN4) presented the c-*met* mutation (c.A3523G, p.His1112Arg) as published previously [8]. As controls, primary PRCC tumors from three patients without c-*met* mutation (Tumor samples UPN1, UPN2, and UPN3) were included in the study for immunostaining experiments.

### 2.2. Generation and Characterization of c-Met-Mutated iPSC

Cellular programming was performed using the patient’s bone marrow mononuclear cells after her informed consent and the approval of Inserm ethical committee. Hereditary PRCC with c-*met*-mutated iPSC *(met*-IPSC) lines were generated using Sendaï virus-mediated gene transfer of the four Yamanaka factors (Figure S1A). As a control we used iPSC generated from bone marrow mononuclear cells of a normal donor. Flow cytometric analysis demonstrated that the majority of *met*-IPSC as well as control iPSC co-expressed SSEA4 and TRA-1-60 (91.3% and 89.8% respectively) markers (Figure S1B,C) as expected for human pluripotent stem cells. In vivo teratoma induction assay in NSG recipient mice revealed differentiation towards all three germ layers (Figure S1D) such as neural rosettes (ectoderm), immature cartilage (mesoderm), and glandular epithelia (endoderm). The same results were obtained using control iPSCs (Figure S1E). These data demonstrated the successful generation of fully reprogrammed *met*-IPSC using this technology.

### 2.3. Generation and Characterization of c-Met-Mutated iPSC Aggregates

To demonstrate that hereditary PRCC with c-*met*-mutated iPSC aggregates (*met*-IPSC-A) can lead to formation of embryoid bodies spontaneously, we cultured *met*-IPSC-A under ultra-low attachment conditions. First, *met*-IPSC-A were characterized using immunocytochemistry for expression of phospho-Met and Brachyury. As shown in Figure S2A–D, these experiments demonstrated that both control and *met*-IPSC-A contained cells expressing phosphorylated c-Met protein (phospho-Met) and expressing Brachyury at 1 and 6 days (Figure S2C,D). Of note, the level of phospho-Met expression was higher in both control and *met*-IPSC-A culture as compared to the monolayer counterparts (Figure S2B). *met*-IPSC-A were then further characterized using immunocytochemistry for expression of kidney related markers SIX2 (SIX homeobox 2, a marker of kidney progenitors) and PODXL (podocalyxin-like, a marker of glomerular podocytes) as described [9,10]. Immunocytochemistry analyses showed the detection of both SIX2- and PODXL-positive cells at day 3 and day 6 (Figure S3A–C). These data demonstrate that aggregate formation strongly favors the emergence of double-positive SIX2 and PODXL expressing kidney progenitors from both control and *met*-IPSC.

### 2.4. Evaluation of a 3D Culture System for Induction of Kidney Differentiation from Met-IPSC 

We performed a 3D culture system protocol to induce kidney differentiation for 12 days. (Figure S3D,E). 3D culture system was compared with the monolayer culture (day 6) of both control and *met*-IPSC to evaluate the efficiency of differentiation into kidney at day 12 (Figure S4A). The expression of kidney markers von Hippel-Lindau (VHL) and PODXL was determined by immunocytochemistry. As seen in Figure S4B, the 3D culture system outperformed the monolayer conditions in terms of level of VHL and PODXL expression. When only a weak expression of both proteins is observed in cells cultured in monolayers, a strong signal is noticed in cells grown in 3D cultures in both groups (Figure S4C). This result indicates that 3D cultures surpassed monolayers as they robustly enabled the generation of VHL- and PODXL-positive cells.

### 2.5. Characterization of Embryoid Bodies Containing Cells with Kidney Markers

**Figure 1:**
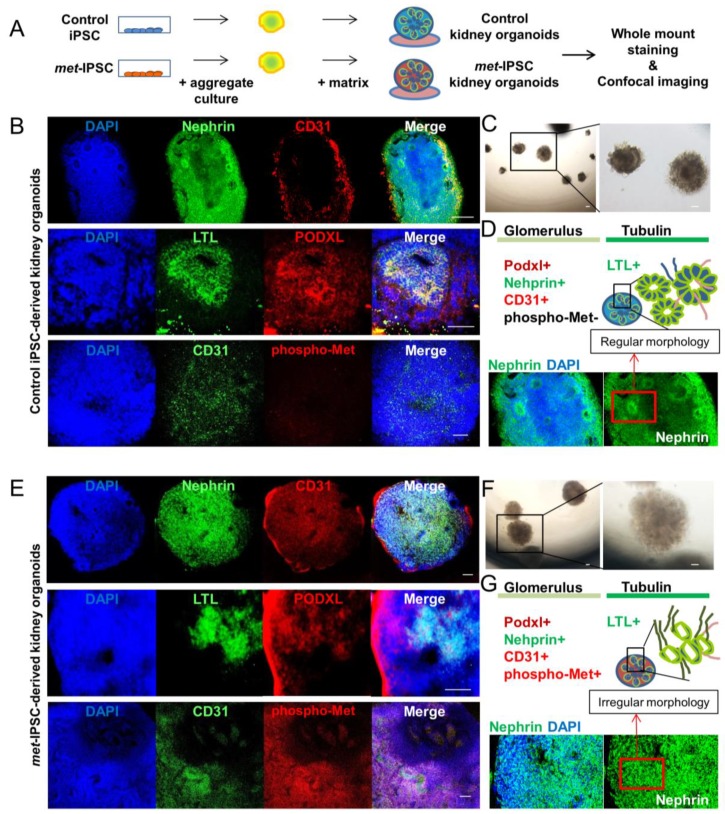
Embryoid bodies with kidney markers from control or *met*-IPSC in 3D culture. (**A**) Schematic representation of the generation of iPSC-derived structures from control or *met*-IPSC. (**B**) Confocal analysis and whole-mount immunostaining for Nephrin, CD31, LTL, PODXL, phospho-Met, and DAPI in control iPSC-derived kidney embryoid bodies showing nephron structure. Scale bar: 100 μm. (**C**) Optical image of control iPSC-derived kidney embryoid bodies at day 12 showing the formation of tubule structures. Scale bar: 100 μm. (**D**) Schematic representation of glomerulus and tubule markers with nephron structure. (**E**) Confocal analysis and whole-mount immunostaining for Nephrin, CD31, LTL, PODXL, phospho-Met, and DAPI in *met*-IPSC-derived kidney embryoid bodies. Scale bar: 100 μm. (**F**) Optical image of *met*-IPSC-derived embryoid bodies at day 12 showing the formation of tubule structures. Scale bar: 100 μm. (**G**) Schematic representation of glomerulus and tubule markers with nephron structure. Note: The *met*-IPSC-derived vesicles (**E**) present an irregular morphology as compared to controls (**B**).

To investigate whether we can generate kidney-like structures from the iPSC-A using our 3D culture system, control iPSC-A as well as *met*-IPSC-A were plated on 24- or 96-well Geltrex-coated dishes or 8-well culture chambers and cultured for another 6 days (Figure S5A). At 12 days whole-mount immunostaining was performed using antibodies against phalloidin (a cytoskeleton marker) and PODXL. After 12 days of differentiation, we obtained two types of structures based on their 3D shape, designed as “cup-like embryoid bodies” type (Figure S5B,D,F) and a “fusion embryoid bodies” type (Figure S5C,E,G). Whole-mount immunostaining revealed that in the “cup-like embryoid bodies” with cavitation, PODXL expression was markedly detected in the center and periphery of the embryoid bodies whereas phalloidin expression was restricted to the periphery (Figure S5B,D). In the “fusion embryoid bodies” type structures we detected strong expression of both PODXL and phalloidin throughout (Figure S5C,E). These results indicate that renal differentiation of *met*-IPSC could be assessed within 2 weeks with the formation of specific kidney embryoid bodies expressing PODXL glomerular marker. We then performed kidney embryoid bodies from both control and *met*-IPSC to compare the expression of glomerular or tubular markers. As seen in Figure 1, our embryoid bodies markedly expressed the glomerulus-specific marker Nephrin, the endothelial marker CD31, phospho-Met and the lotus tetragonolobus lectin (LTL) a marker of proximal tubule epithelial cells (Figure 1B,E). Interestingly, glomerulus and tubulin structure in control kidney embryoid bodies seems to be spatially organized (Figure 1B,D), as shown by staining distribution, and shape of the embryoid bodies than the *met*-IPSC-derived kidney embryoid bodies (Figure 1E,G).

### 2.6. Structural and Functional Evaluation of Embryoid Bodies

**Figure 2:**
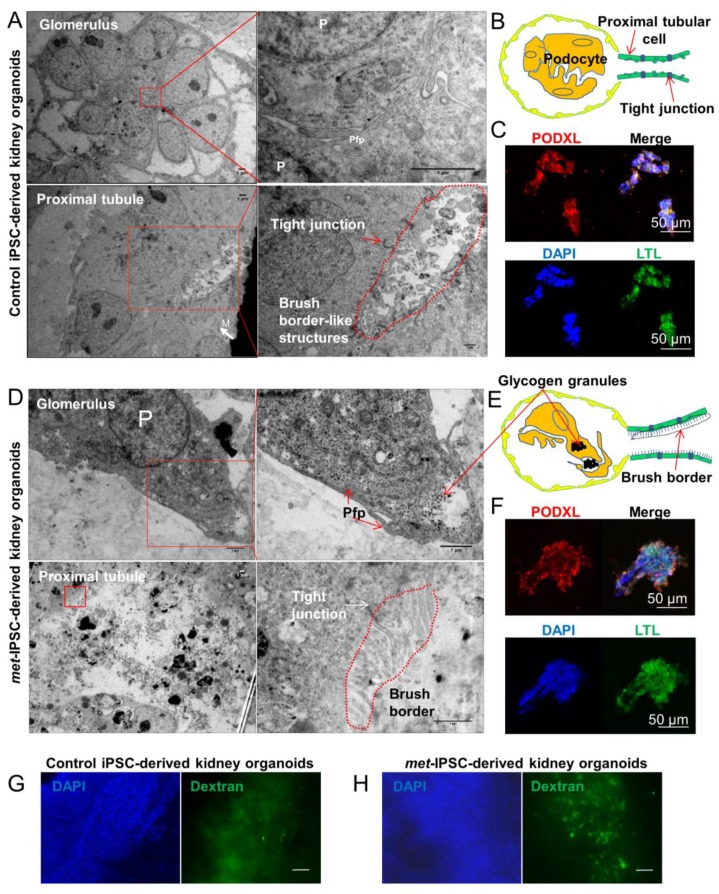
Ultrastructure and immunohistochemistry analyses by confocal microscopy. (**A**–**C**) Representative electron microscopy images of glomeruli and tubules of control and *met*-IPSC-derived embryoid bodies (EBs) showing podocyte-like cells (P), podocyte-like foot process (Pfp), mitochondria (M) and brush borders. The immunohistochemistry analysis in paraffin cuts reveals both PODXL and LTL positivity in embryoid bodies. Scale bar: 50 μm. (**D**–**F**) Representative electron microscopy images glomeruli and tubules of *met*-IPSC-derived kidney embryoid bodies revealing podocyte-like cells (P), podocyte-like foot process (Pfp), tight junctions, and typical brush border structures. Immunohistochemistry analysis in paraffin cuts by confocal imaging showed embryoid bodies structures co-expressing PODXL and LTL. Scale bar: 50 μm. As compared to control iPSC-derived embryoid bodies, the presence of glycogen granules(*) were noted (**D**). (**G**–**H**) Evaluation of functional analysis of proximal tubules structures in kidney embryoid bodies by using Dextran uptake assays. Embryoid bodies were incubated for 48 h in the presence of Dextran–Alexa 488 followed by analysis using wide-field microscopy. Dextran uptake was seen in normal (**G**) as well as in *met*-IPSC-derived embryoid bodies (**H**) with a more intense uptake in the latter structures (see text). Scale bar: 100 μm.

**Figure 3:**
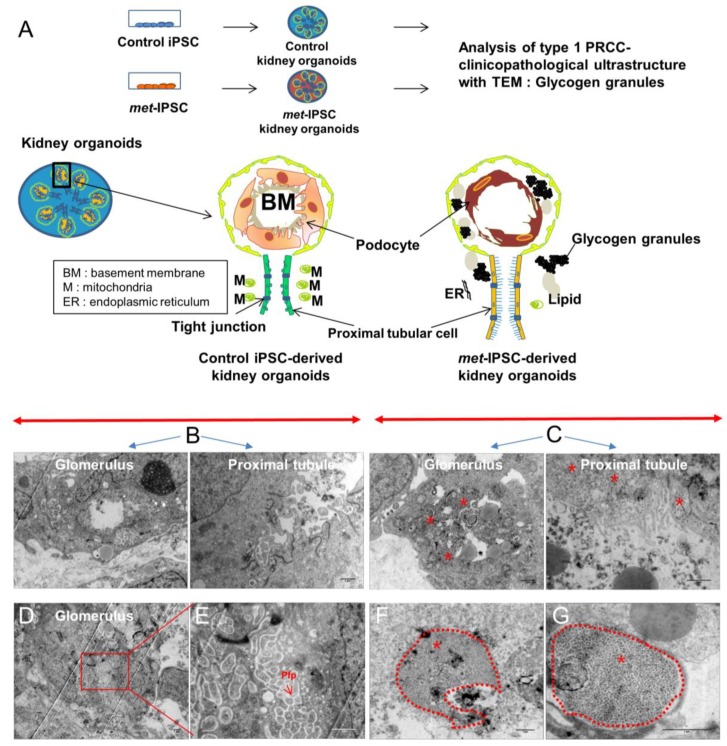
Ultrastructural analysis of kidney embryoid bodies derived from control or c-*met*-mutated iPSC. (**A**) Schematic representation of the experimental protocol used. (**B**,**C**) Representative electron microscopy images of glomerulus and tubules structures of control or c-*met*-mutated iPSC-derived kidney embryoid bodies. (**D**,**E**) Representative electron microscopy images cytoplasm region of control iPSC-derived kidney embryoid bodies. (**F**,**G**) Representative electron microscopy images cytoplasm region of c-*met*-mutated iPSC-derived kidney embryoid bodies, podocyte-like cells. Glycogen granules (*).

**Figure 4:**
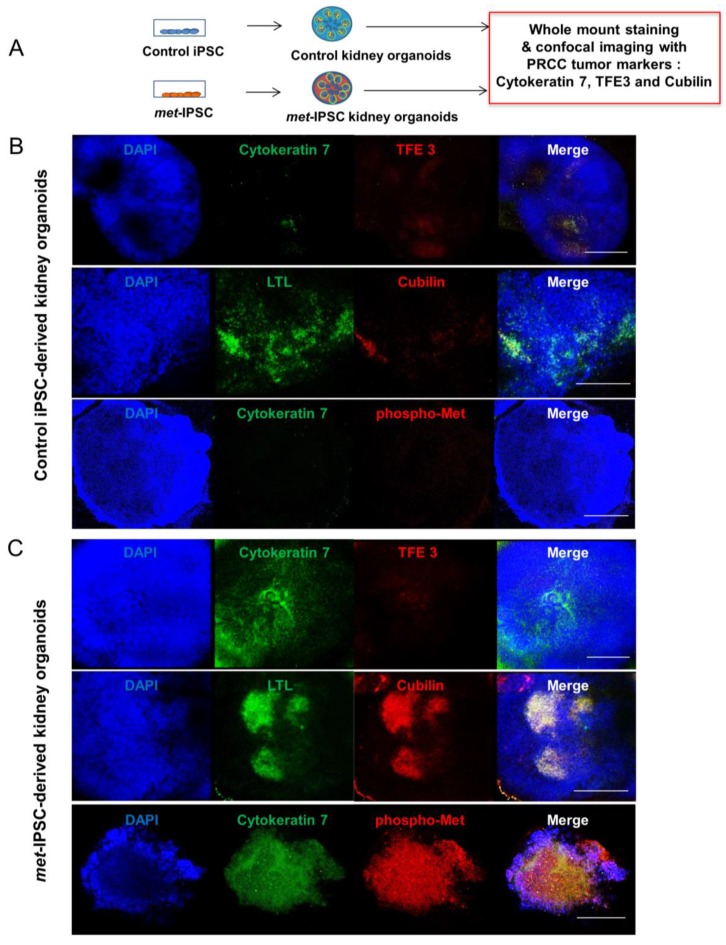
Characterization of *met*-IPSC-derived kidney embryoid bodies. (**A**) Schematic representation of the generation of control or *met*-IPSC-derived kidney embryoid bodies and characterization of clinicopathological cancer markers cytokeratin 7, TFE3, and Cubilin. (**B**) Whole-mount staining for Cytokeratin 7, TFE3, LTL, Cubilin, and DAPI in control iPSC-derived kidney embryoid bodies. Scale bar: 500 μm. (**C**) Whole-mount staining for Cyto-keratin 7, TFE3, LTL, Cubilin, and DAPI in *met*-IPSC-derived kidney embryoid bodies. Scale bar: 500 μm.

Spatiotemporal organization and ultrastructure of embryoid bodies were analyzed by performing transmission electron microscopy (TEM) (Figure 2A,D, Figure 3 and Figure S6). As can be seen in Figure 2A, these experiments clearly showed ultrastructures corresponding to the generation of tight junctions (Figure 2A) and brush border-like structures expected to be seen in proximal tubules (Figure 2A). Interestingly, the brush borders detected by TEM in *met*-IPSC-derived kidney embryoid bodies were well developed as compared to brush borders from control iPSC-derived kidney embryoid bodies (Figure 2A,D and Figure S6D). In the same sections used for TEM, these structures have been found to express PODXL and LTL, confirming the presence of glomerular and tubular cells derived from *met*-IPSC or control iPSC (Figure 2C,F). Interestingly, large amounts of glycogen granules were observed in *met*-IPSC-derived kidney embryoid bodies (Figure 2D, and Figure 3C,F,G). We then analyzed the functionality of proximal tubule structures by using a dextran uptake assay in embryoid bodies at day 12. These assays demonstrated the selective uptake of dextran–Alexa488 from the media after 48 h of exposure in kidney embryoid bodies (Figure 2G,H). These findings demonstrated that the embryoid bodies structures obtained in our experiments corresponded to kidney cells generated in vitro from *met*-IPSC and control iPSC. Next, by using kidney embryoid bodies model, we asked whether biomarkers expressed in kidney cancer, could be expressed in *met*-IPSC-derived kidney embryoid bodies. For this purpose, we performed whole-mount immunostaining using cytokeratin 7 (Cy7) [11], TFE3, and Cubilin antibodies, well-established markers of PRCC (Figure 4A). As can be seen in Figure 4B, cytokeratin 7 and Cubilin expression were detected in *met*-IPSC-derived kidney embryoid bodies whereas TFE3 expression was similar to that observed in control iPSC-derived kidney embryoid bodies (Figure 4B,C). As compared with control iPSC-derived kidney embryoid bodies, phospho-Met signal was strongly detected in *met*-IPSC-derived kidney embryoid bodies (Figure 4B,C). Next, to explore the genomic consequences of constitutionally active c-*met* signaling pathway in kidney cells and to explore the genomic circuitry activated in kidney embryoid bodies, we performed a global transcriptome analysis in the *met*-IPSC-derived kidney embryoid bodies compared to gene profile of controls. As seen in Table S2 and Figure S7A, 244 genes were found up-regulated and 71 down-regulated. Gene set enrichment analysis (GSEA) revealed an enrichment of targets involved in kidney development specifically in *met*-IPSC-derived kidney sample such as regulation of epithelial cell differentiation involved in kidney development (Normalized Enrichment Score (NES = +1.55, *p* < 0.001, Figure S7B), renal tubule development (NES = +1.51, *p* < 0.001, Figure S7C), and positive regulation of kidney development gene set (NES = +1.48, *p* < 0.001, Figure S7D). Also, GSEA analysis revealed implication of pathophysiological process between these two kidney sample conditions with an enrichment of renal cell carcinoma (NES = +1.40, *p* < 0.001, Figure S7E) and aging kidney gene sets (NES = +1.67, *p* < 0.001, Figure S7F) specifically in *met*-IPSC-derived kidney embryoid bodies compared to control counterparts.

### 2.7. Kidney Capsule Transplantation Experiments of Met-IPSC-Derived Embryoid Bodies

**Figure 5:**
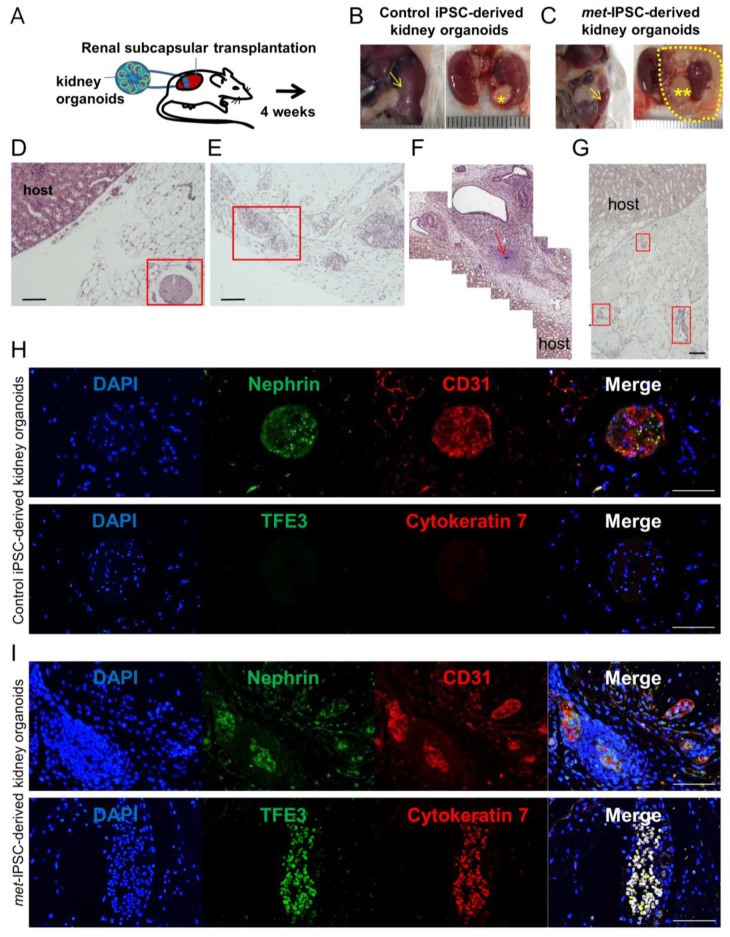
Analysis of tumors generated by transplantation of kidney embryoid bodies. (**A**) Experimental protocol used. (**B**,**C**) Macroscopic aspect of kidney NSG mouse 1 month after transplantation of control (*) or *met*-IPSC-derived kidney embryoid bodies (**), mouse kidney (arrow). (**D**,**H**) Pathological analysis of tumors generated by transplantation of control kidney embryoid bodies at 1 month showing the presence of normal embryoid bodies-like structures expressing kidney differentiation markers. (**E**–**G**,**I**) Pathology of c-*met*-mutated tumors after transplantation of *met*-IPSC-derived kidney embryoid bodies at 4 weeks, showing disorganized structures with expression of kidney cancer markers TFE3 and cytokeratin 7. Immature cartilage (arrow). Scale bar: 100 μm.

To determine if *met*-IPSC-derived structures can recapitulate the features observed in vivo, we transplanted them under the kidney capsule of NSG mice. At 4 weeks post-transplant, mice were sacrificed, and harvested structures were subjected to histopathological and immunohistochemistry analyses (Figure 5A). We observed that *met*-IPSC-derived embryoid bodies generated larger teratoma-like tumors compared to controls (Figure 5B,C). Histopathological analyses using H&E staining revealed the presence of immature cartilage in tumors of *met*-IPSC group (Figure 5F). Moreover, disorganized structures were observed in the cross-sections of *met*-IPSC-derived embryoid bodies-like structures (Figure 5E,G). In contrast, control iPSC showed normal structures (Figure 5D). Immunohistochemistry analysis using Nephrin and CD31 antibodies (Figure 5H,I) demonstrated the presence of podocytes and endothelial cells in both groups. Notably, expression of PRCC markers cytokeratin 7 and TFE3 (Figure 5H,I) were found markedly increased in *met*-IPSC-derived embryoid bodies as compared to controls. Altogether, these data suggest that a PRCC-like phenotype can be recapitulated in vivo using *met*-IPSC-derived embryonic structures.

### 2.8. Hereditary PRCC with c-Met-Mutated iPSC Aggregates Reproduce Molecular Features of Human PRCC

**Figure 6:**
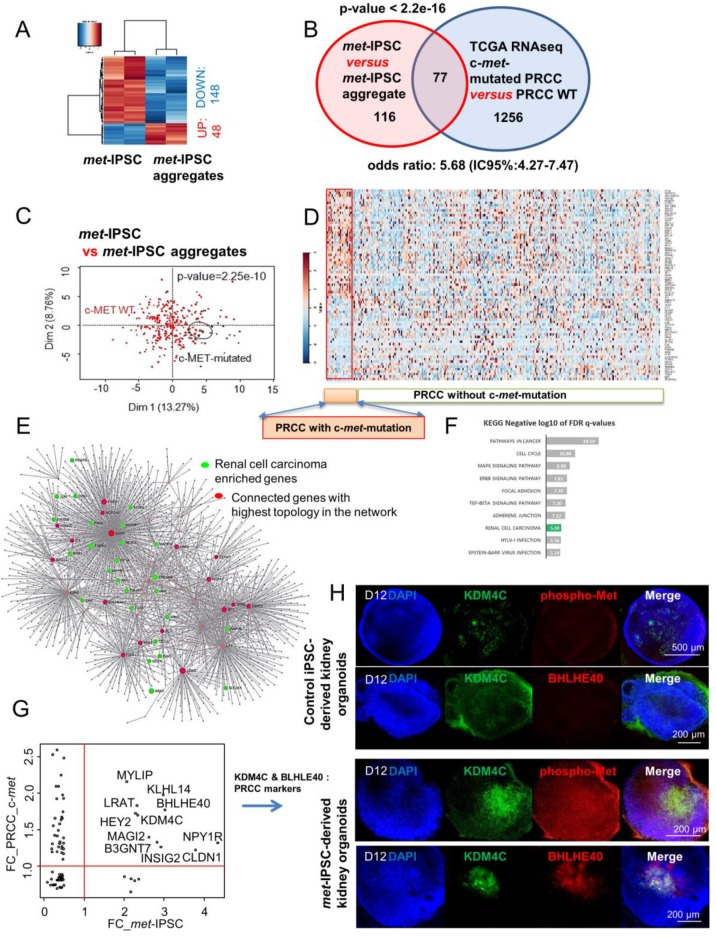
Transcriptome analyses of *met*-IPSC aggregates. (**A**) Expression heatmap (Euclidean distances) of differential expressed genes found between *met*-IPSC (monolayer culture) and *met*-IPSC aggregates. (**B**) Venn diagram of the meta-analysis between *met*-IPSC and PRCC patient analysis, *p*-value of the *met*-IPSC signature in PRCC expression profile was calculated by hypergeometric test of Fisher. (**C**) Principal component analysis performed with meta-analysis gene intersection (77 genes) on PRCC tumor samples (Z-scores RNA-seq V2), *p*-value was calculated by correlation of the group discrimination of the first principal component. (**D**) Expression heatmap performed with meta-analysis gene intersection (77 genes) on PRCC tumor samples. (**E**) protein-protein interaction network built with the projection of 77 meta-analysis genes on innateDB interaction database: red genes represent connected genes with the highest topology in the network, green genes represent enriched gene during Kyoto Encyclopedia of Genes and Genomes (KEGG) inference and related to the Renal cell carcinoma. (**F**) Bar plot representing negative logarithm base 10 of False Discovery Rate (FDR) q-values found during functional inference of KEGG database on meta-analysis protein-protein interaction (PPI) network. (**G**) Concordance scatterplot of fold-change (FC) found during meta-analysis of transcriptome. The x-axis shows FC of the genes found in *met*-IPSC transcriptome study and the y-axis shows the FC of genes found in analysis of PRCC RNA-seq study. The genes found to be expressed > 1 FC are shown by their gene symbols. (**H**) Whole-mount immunostaining for KDM4C (Lysine demethylase 4C), phospho-Met and DAPI in kidney embryoid bodies.

To determine the gene-expression pattern of *met*-IPSC, we performed transcriptome analysis on both *met*-IPSC and *met*-IPSC-A. Supervised analysis between the 2 classes of culture *met*-IPSC versus *met*-IPSC-A by ranking products algorithm enabled us to identify 196 differentially expressed gene probes (Table S3). 148 of these genes were found to be down-regulated in *met*-IPSC-A as compared to *met*-IPSC. A small fraction of them (*n* = 48) were found to be up-regulated in *met*-IPSC-A as compared to *met*-IPSC (Figure 6A and Tables S3 and S4). In parallel, we investigated analysis of TCGA consortium Next Generation Sequencing dataset of PRCC tumor samples from the most recent cohort study of transcriptome with mutation sequence status that was available [12]. This allowed us to stratify the transcriptome of PRCC by their c-*met* mutation status: 23 mutations were found to be present in 21 patients (Table S5). Machine learning supervised by c-*met* status performed on PRCC RNA-seq samples allowed to characterize 1333 predictive genes with a minimum error of misclassification (data not shown). Meta-analysis between *met*-IPSC signature and PRCC signature revealed a significant enrichment of *met*-IPSC profile as predictive of c-*met*-mutated PRCC tumor status (Fold of enrichment: 5.68; *p*-value < 2.2 × 10^−16^, Figure 6B). Unsupervised principal component analysis performed with *met*a-analysis intersection genes (77 genes, Table S4) on the transcriptome of PRCC tumor patients confirmed a significant stratification of these tumor samples taking into account their c-*met* mutation status (*p*-value = 2.25 × 10^−10^, Figure 6C). Expression heat map performed on PRCC tumors samples with the 77 *met*a-analysis intersection genes (Figure 6D) revealed a distinct expression pattern of these genes in PRCC patients that carried c-*met* mutations as compared to others; also the proportion of up-regulated and down-regulated genes is even in this subgroup of patients. These results suggest that the gene-expression profile of *met*-IPSC-A is an efficient model predictor of PRCC tumor stratification taking into account their c-*met* mutation status. To understand the influence of *met*-IPSC-A signature study in the PRCC, a protein-protein interaction (PPI) network was built with extraction of the protein interactions from innateDB database concerning the 77 genes found at the intersection of the *met*a-analysis (Table S4). This interaction network analysis allowed to build a principal network comprising 65 seeds, 1713 nodes and 2204 edges (Figure 6E). Analysis of topology network importance revealed central connectivity of key molecules such as EGR1 and other stem cell related molecules (EZH2, NOTCH2, GLI3) (red nodes in Figure 6E). Functional inference performed on this interaction network performed with projection of Kyoto Encyclopedia of Genes and Genomes (KEGG) pathway database revealed significant implication of genes already involved in renal carcinoma (green nodes in Figure 6E, and green bar in Figure 6F). Among these 26 kidney cancer genes, HGF which is the ligand of c-*met* was predicted to be connected in this network (Figure 6E). Fold-change concordance analysis between *met*-IPSC-A model and PRCC tumors revealed 11 up-regulated markers with c-*met* mutation status (Figure 6G). Among these 11 genes some were found with high connectivity on the previous interaction network such as KDM4C, which is implicated in chromosomal aberrations found in tumors and BHLHE40 an important regulator of circadian rhythms (ARNTL1 partner) and cell differentiation (Figure 6G). This network analysis confirmed by inference that the use of iPSC-aggregates gene signature during *met*a-analysis can efficiently predict c-*met* status stratification of PRCC tumors. Furthermore, KDM4C expression was validated in our *met*-IPSC-derived embryoid bodies as we observed a co-expression of KDM4C and phospho-Met in *met*-IPSC-derived kidney embryoid bodies (Figure 6H, lower panels). Control iPSC-derived kidney embryoid bodies exhibited low levels of KDM4C expression which did not overlap with that of phospho-Met (Figure 6H, upper panels). These data demonstrate that *met*-IPSC-derived kidney embryoid bodies recapitulate some molecular features of human PRCC and could be of major interest not only to model this hereditary cancer but also to further understand the molecular circuitry downstream of c-*met* during the progression of this hereditary disease.

### 2.9. Validation of the Expression of Markers Detected in c-Met-Mutated iPSC-Derived Kidney Embryoid Bodies in Primary PRCC Tumors

**Figure 7:**
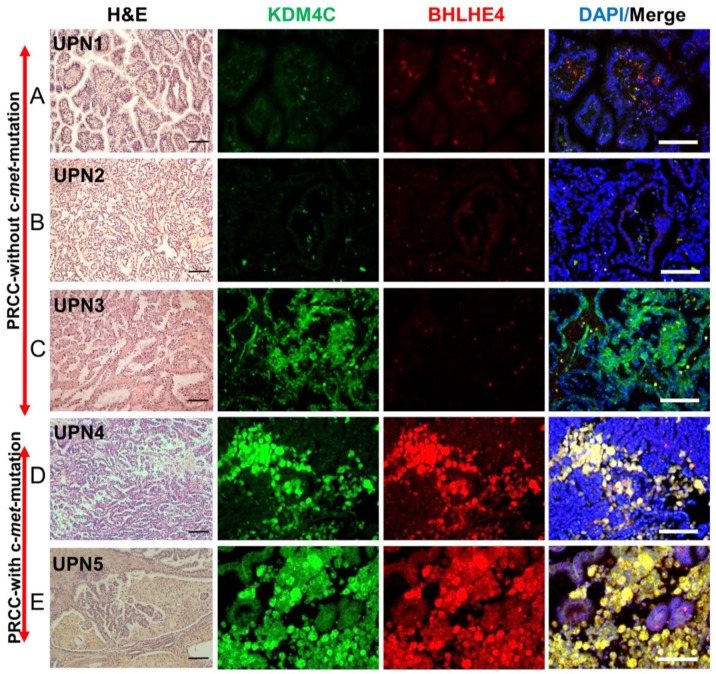
Immunohistochemistry of primary kidney cancer samples from patients without and with c-*met* mutated PRCC**.** Hematoxylin-eosin (H&E) staining and immunohistochemistry for KDM4C, BHLHE40, and DAPI from paraffin-cut primary tumor samples. (**A**–**C**) (UPN1, 2, 3): PRCC without c-*met* mutation. (**D–E**) (UPN4, 5): PRCC with c-*met* mutation (see Table S1). Scale bar: 100 μm.

We next asked whether the candidate genes that we have found overexpressed in *met*-IPSC embryoid bodies were also expressed in primary papillary renal carcinoma. For this purpose, we analyzed 5 primary PRCC, 2 from patients with c-*met* mutation (UPN4 and UPN5) and 3 from patients without c-*met-*mutation (UPN1, UPN2, and UPN3). One of the c-*met*-mutated PRCC sample (UPN5) is from the mother of our patient (Table S1). Among the 11 candidate genes that we have previously identified, we focused on KDM4C and BHLHE40 genes which are known to be expressed in PRCC. To evaluate their level of expression, we performed immunohistochemistry in primary tumors derived from type 1 PRCC with and without and c-*met* mutation using specific antibodies. As seen in Figure 7, KDM4C and BHLHE40 were markedly overexpressed in the c-*met*-mutated type 1 PRCC tumors (UPN4, UPN5, Figure 7D,E) as compared to type 1 PRCC tumors without c-*met* mutation (UPN1, UPN2, UPN3, Figure 7A–C). These results strongly suggested that the findings observed in *met*-IPSC-derived kidney embryoid bodies are truly representative of tumor marker expression in the primary tumors from patients with PRCC.

### 2.10. Drug Toxicity Assays

**Figure 8:**
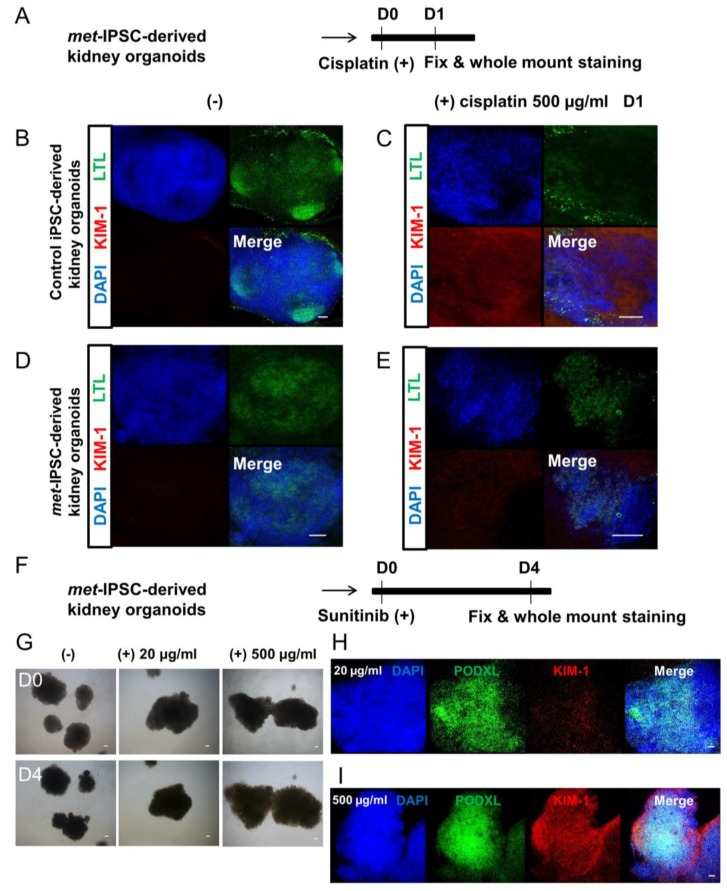
**Figure 8.** Drug toxicity experiments. (**A**) Schematic representation of cisplatin toxicity experiments in *met*-IPSC kidney embryoid bodies. (**B**–**C**) Representative whole-mount immunostaining for LTL, KIM-1 and DAPI in control iPSC-derived kidney embryoid bodies treated with cisplatin (500 µg/mL) (right panels). (**D**–**E**) Representative whole-mount immunostaining for LTL, KIM-1 and DAPI in *met*-IPSC-derived kidney embryoid bodies treated with cisplatin (500 µg/mL) (right panels). (**F**) Schematic of drug Sunitinib toxicity test process of *met*-IPSC-derived kidney embryoid bodies. (**G**) Photograph of *met*-IPSC-derived kidney embryoid bodies in 96 well plate. (**H**) Representative whole-mount immunostaining for PODXL, KIM-1 and DAPI in *met*-IPSC-derived kidney embryoid bodies treated with Sunitinib (20 µg/mL). (**I**) Representative whole-mount immunostaining for PODXL, KIM-1 and DAPI in *met*-IPSC-derived kidney embryoid bodies treated with Sunitinib (500 µg/mL), Scale bar: 100 μm.

To test whether our embryoid bodies could be used to study kidney toxicity in vitro, we tested two different drugs on our *met*-IPSC-derived kidney embryoid bodies (Figure 8). After 12 days of differentiation, embryoid bodies were treated for 3 days with two doses of Sunitinib (either 20 µg/mL or 500 µg/mL), a commonly used tyrosine kinase inhibitor in PRCC therapy. Upon treatment, we analyzed the presence of the Kidney Injury Molecule-1 (KIM-1), a biomarker up-regulated in the proximal tubules following acute kidney injury. As seen in Figure 8H,I, KIM-1 was detected at low level in structures treated with low dose of Sunitinib (20 µg/mL) whereas a higher dose of Sunitinib (500 μg/mL) resulted in higher levels of KIM-1 (Figure 8H,I). Similarly, *met*-IPSC-derived kidney-like structures treated with cisplatin harbored KIM-1 up-regulation albeit to lesser degree than that observed in control iPSC-derived structures (Figure 8B–E). These data showed that *met*-IPSC-derived embryoid bodies with kidney features could be used to test the nephrotoxicity and perhaps evaluate the therapeutic efficacy of novel candidate drugs in vitro.

## 3. Discussion

Germline mutations at the origin of family cancers represent a major challenge in oncology as there are no experimental models to study the future cancer development. *c-met*-mutated PRCC represents a major form of hereditary kidney cancer [13] among kidney cancers which are the seventh most common malignancies in the United States [14]. PRCC includes tumors with indolent, multifocal presentation, and solitary tumors with an aggressive, highly lethal phenotype.

We describe here the first model to our knowledge, of a *met*-IPSC-derived patient-specific embryoid bodies reminiscent of PRCC. The first step of our experiments consisted on the design of experimental conditions allowing efficient and reproducible generation of differentiation towards embryoid bodies. To this end, we have used 3D culture of iPSC in the presence of E8 medium. As can be seen in Figure 1F and Figure S3E, *met*-IPSC can differentiate spontaneously into embryoid body-like structures under these 3D culture conditions. Indeed, the *met*-IPSC-derived structures were found to express kidney cell markers such as PODXL+, Nephrin+ and LTL+ [10,15].

Previous kidney embryoid bodies studies involved chemically defined protocols under monolayer culture conditions at the initial stage for human embryonic stem cells [16] or human fibroblast-derived iPSC [16,17] because these conditions allow the control of long-term clonal growth and multilineage differentiation of the pluripotent cells. The large-scale expansion of these cells is relatively easy under monolayer culture conditions. Despite these advantages, such cultures lack cell–cell and cell–matrix interactions and fail to mimic cellular functions and signaling pathways occurring naturally in 3D culture conditions. One of the most efficient kidney embryoid bodies differentiation includes indeed a combination of monolayer and three-dimensional (3D) cultures [17]. The monolayer culture techniques require however, the use of various chemically defined factors to induce kidney differentiation. The method used here addressed these limitations by the occurrence of a self-organization during spontaneous development in vitro and circumvented the disadvantages of monolayer cultures requiring several stages of differentiation without induction of sufficient cell–cell and cell–matrix interactions. 3D culture conditions favoring these interactions generate the potential of differentiation into three germ layers via cellular microenvironment like embryoid bodies (EBs). Such EB culture conditions have been used to generate heart, kidney, and liver embryoid bodies [18]. Finally, we have found that 3D culture conditions induced higher expression of VHL protein as compared to monolayer cultures creating a favorable condition for kidney embryoid body generation (Figure S4B,C). It is known that overexpression of VHL induces kidney cell differentiation through the integration of cell–cell and cell–matrix signaling in 786-O cells [19].

We have then used this aggregate culture conditions to determine the possibility of generation of kidney embryoid bodies using *met*-IPSC and control iPSC. As can be shown in Figure S2B–D, the comparison of monolayer cultures and aggregate cultures revealed the overexpression of phospho-Met in both *met*-IPSC and control iPSC. These phospho-Met overexpressing aggregates generated kidney embryoid bodies as demonstrated by the expression of PODXL in confocal microscopy experiments (Figure 1E, Figure 2F and Figure S5B–G) and that of Nephrin and LTL. As shown in Figure S5, we have found that *met*-IPSC aggregated spontaneously generating fusion structures undergoing cavitation. It is known that the cavitation process is essential for providing a free epithelial surface for the morphogenetic movement of epiblastic cells during the subsequent formation of a primitive streak [20]. This fusion could occur through the use CXCR4/CXCL12 axis, which is known to be essential for kidney vasculature development [21]. Indeed, whole-mount immunostaining experiments showed the expression of CD31 in kidney embryoid bodies (Figure 1B,E). To demonstrate the efficient kidney embryoid body generation, we have used transmission electron microscopy experiments. These experiments confirmed, the appearance of kidney structures including glomeruli, podocytes, glomeruli-associated basement membranes and proximal tubule with typical brush border-like structures (Figure 2A,D and Figure S6). Interestingly, in *met*-IPSC-derived kidney embryoid bodies, a well-developed brush border was observed (Figure 2D and Figure S6D) and high numbers of glycogen granules were observed as previously reported in primary tumors [22] (Figure 3C,F,G), demonstrating the reproduction of a type 1 PRCC tumor phenotype in glycogen granules.

The next important question was to determine if some phenotypic cancer marker known to be expressed in human PRCC could be found in *met*-IPSC-derived structures. Cy7 overexpression was found in *met*-IPSC-derived cells as compared to controls (Figure 4). Moreover, to demonstrate the malignant potential of c-*met*-IPSC-derived structures, we have transplanted them as well as control cells, under the kidney capsule of NSG mice. As can be seen in Figure 5, *met*-IPSC-derived cells induced larger tumors as compared to controls and expressed kidney cancer markers TFE3 (transcription factor for immunoglobulin heavy chain enhancer 3) [23] and cytokeratin 7 [24] demonstrating the generation in vivo cancer embryoid bodies from the *met*-IPSC.

We next asked whether a differential gene-expression profiling can be obtained in *met*-IPSC and aggregates during the induction of aggregates as compared to control iPSC-derived cells. This analysis allowed us to identify 196 differentially expressed genes, generating a clear transcriptome signature during the first stages of kidney embryoid bodies differentiation (Figure 6A). Interestingly, 77 of these genes have also been found to be expressed specifically in c-*met*-mutated human kidney carcinoma (Figure 6B) which appeared to be different from the signature observed in PRCC without c-*met* mutation. Most importantly, several of the 11 genes which have been identified as being overexpressed in our *met*-IPSC, have also been overexpressed in primary human c-*met*-mutated PRCC such as KDM4C and BHLHE40 (Figure 7). KDM4C is a member of the Jumonji-domain 2 family encoding a de*met*hylase involved in chromosome segregation [25]. Alteration of KDM4C gene has been shown to occur in renal cell carcinoma [26]. We next asked whether the overexpression of this gene, which has been reported in both our iPSC and primary human PRCC, could be reproduced in our *met*-IPSC-derived kidney embryoid bodies. As can be seen in Figure 6H, KDMC4 expression was clearly seen in phospho-Met-expressing *met*-IPSC as compared to controls. Similarly, the basic helix-loop helix protein BHLHE40 known to be implicated in c-*met* mutated PRCC [12] was found to be overexpressed in *met*-IPSC-derived embryoid bodies (Figure 6H).

Finally, it was of importance to determine if the two markers that we have discovered through the analysis of *met*-IPSC could be truly representative of the primary cancer cells. To this end, we have used primary kidney tumors from 2 patients with c-*met*-mutated PRCC and 3 PRCC patients without c-*met* mutation. The factors KDM4C and BHLHE40 were found to be overexpressed only in the tumors with c-*met* mutation, validating the use of iPSC technology for the analysis of these hereditary cancers. We confirmed that phospho-Met, KDM4C and BHLHE40 were overexpressed in the tumors of two patients with the c-*met*-mutated type 1 PRCC UPN4 and UPN5 (Figure 7D,E). These data demonstrate for the first time, the major interest of the use of iPSC technology to model a hereditary cancer allowing the reproduction of the “hereditary renal cancer in a dish” concept opening the perspective for novel drug testing strategies using simple in vitro tests. From this regard, our first results using Sunitinib and cis-Platinum, demonstrate clearly the feasibility of this approach by testing the expression of the KIM-1 protein a known a marker for kidney proximal tubular damage and toxicity in kidney [27].

The data reported here also show the possibility of using this technology for discovering novel therapies and targets in hereditary cancers. In recent years, major discoveries allowed rapid development of cancer therapies including immunotherapy using checkpoint inhibitors in genito-urinary cancers [28]. More specifically, in PRCC, several novel targetable signaling pathways have been discovered such as VEGFR, mTOR, FGFR, RET, KIT and AXL [28]. The use of the iPSC-derived organoid technology could open unprecedented opening in terms of personalized medicine in this setting, allowing the efficacy of targeting these signaling pathways alone or in combination. More specifically, the c-Met-mutated iPSC organoid technology that we describe, could be also a major experimental platform for testing the efficiency of novel and future MET inhibitors in cancer therapy [29].

In conclusion, we demonstrate in this work for the first time that c-*met*-mutated hereditary kidney cancer can be modeled in vitro using patient-derived iPSC. This could be achieved by a simple and reproducible 3D culture system leading to generate embryoid bodies without chemically defined induction steps. These embryoid bodies-like structures could be used for drug toxicity testing. We show that *met*-IPSC-derived structures have the potential to generate the gene profiling similar to that found in primary c-*met*-mutated PRCC, reproducing the expression of several genes known to be expressed in primary cancer cells. Among these, KDM4C is a histone demethylase and its overexpression has been shown in several cancers including breast, colon and prostate cancers [30]. In PRCC, CNV of this gene has previously been shown [26]. The pathophysiological role of KDM4C overexpression in c-*met*-mutated PRCC suggest that this pathway can be druggable, as small molecules inhibiting KDM4C are currently been developed [31]. The BHLHE40 (DEC1/Stra13) is a well-known basic helix-loop transcription factor and has been shown to play major roles in cell proliferation, circadian rhythm, tumor progression [32] and has been shown to be overexpressed in papillary renal cell carcinoma [33]. This work confirms that the discovery of the potential involvement of these pathways is possible using cancer embryoid bodies derived from iPSC lines established from hereditary cancers. These findings could open novel perspectives for drug-screening as well as future precision-medicine strategies in hereditary cancers and could be applied to other hereditary cancers as previously shown in BRCA1-mutated breast [5] and Li-Fraumeni-syndrome [3] and RET-mutated [2] cancers. Finally, in the setting of healthy persons presenting a hereditary cancer-risk mutation, these results could pave the way for the future use of this technology to generate predictive strategies.

## 4. Materials and Methods

### 4.1. Key Resource Table


**Reagent or Resource**

**Source**

**Identifier**
Antibodies

BrachyuryAbcam (Cambridge, UK)ab140661 PODXLAbcam (Cambridge, UK)ab178566LTLClinisciences (Nanterre, France)FL-1321CubilinAbcam (Cambridge, UK)ab191073SIX2Euromedex (Souffelweyersheim, France)11562-1-APCD31Abcam (Cambridge, UK)ab24590TFE 3Abcam (Cambridge, UK)ab179804Cytokeratin 7Abcam (Cambridge, UK)ab9021NephrinAbcam (Cambridge, UK)ab85379KIM-1Bio-Techne (Minneapolis, USA)NBP1-76701PhalloidinInvitrogen (Carlsbad, USA)A12381Phospho c-MetOzyme (Saint-Cyr-l’École, France)3077SKDM4CAbcam (Cambridge, UK)LS-C114684-100VHLBio-Techne (Minneapolis, MN USA)SC-5575BHLHE40Abcam (Cambridge, UK)ab70723DAPISigma–Aldrich (St. Louis, MO, USA)D9542
**Chemicals, Peptides, and Recombinant Proteins**


Essential 8 basal medium Thermo Fisher Scientific (Waltham, MAUSA)A1516901Essential 8 supplementThermo Fisher Scientific (Waltham MA, USA)A1517101Geltrex^®^ LDEV-Free Reduced Growth Factor Basement Membrane MatrixThermo Fisher Scientific (Waltham, MA, USA)A1413202
**Reagent or Resource**

**Source**

**Identifier**
Experimental Models: Cell Lines

Human iPSC: PB33Human (Villejuif, France)-Human c-*met* mutated iPSC: PB56Human (Villejuif, France)-Cis-platinumSigma–Aldrich (St. Louis, MO, USA)P4394Osmium tetroxide solutionSigma–Aldrich (St. Louis, MO, USA)75632Glutaraldehyde grade ISigma–Aldrich (St. Louis, MO, USA)G5882Experimental Models: Organisms/Strains

Mouse: NOD.Cg-Prkdcscid Il2rgtm1Wjl/SzJ
N/A
**Software and Algorithms**


ImageJ (Public domain, USA)



### 4.2. Generation of Hereditary PRCC with c-Met-Mutated iPSC

Bone marrow mononuclear cells (BMNC) were obtained with the informed consent of the patient according to the Declaration of Helsinki and the approval of the Inserm ethical committee which gave approval for the use of iPSC generation from hereditary cancers (PP 1301 14 January 2014). Cell programming was performed using previously reported procedures [2,34].

### 4.3. Flow Cytometry

Control iPSC or *met*-IPSC colonies were collected using 1 mg/mL collagenase IV (Thermo Fisher Scientific, Waltham, MA, USA). A single cell suspension was obtained by incubation in Enzyme Free Cell Dissociation Buffer (Thermo Fisher Scientific, Waltham, MS, USA) followed by mechanical trituration. 100,000 cells were stained in 10 µL phosphate-buffered saline (PBS) supplemented with 1 µL of primary antibodies raised against SSEA4 conjugated to BD Horizon™ V450, SSEA3 conjugated to Phycoerythrin and TRA-1-60 conjugated to Alexa Fluor™ 647 (all of them from BD Biosciences, San Jose, CA, USA) for 30 min at 4 °C. Cells were then washed with PBS and analyzed using a MACSQuant flow cytometer (MiltenyiBiotec, Bergisch Gladbach, Germany).

### 4.4. Teratoma Assay

Animal experimentation was performed according to French regulations. Protocols were approved by the Ethical Committee for Animal Experimentation (CEEA n°26) under the agreement number 2015-012-534. Experiments were performed using female mice aged 6 to 7 weeks old. Control iPSC or *met*-IPSC were collected by collagenase IV treatment (Thermo Fisher Scientific, Waltham, MA, USA). 2 × 10^6^ cells were resuspended in 150 µL of Geltrex^TM^:DMEM/F12 (1:1) and were subcutaneously injected into the rear leg of NSG mice (NOD.Cg-Prkdcscid Il2rgtm1Wjl/SzJ). After 8 or 12 weeks, teratomas were isolated and processed for histological analysis.

### 4.5. Cell Culture and Generation of Cell Aggregates

Control and *met*-IPSC were maintained on Geltrex (Stem Cell Technologies, Inc, Vancouver, Canada) coated flat culture dish in E8 media (Stem Cell Technologies, Inc, Vancouver, Canada) contained DMEM/F12, L-ascorbic acid-2-phosphate magnesium (64 mg/L), sodium selenium (14 μg/L), FGF2 (100 μg/L), insulin (19.4 mg/L), NaHCO_3_ (543 mg/L) and transferrin (10.7 mg/L), TGFβ1(2 μg/L) or NODAL (100 μg/L). Osmolarity of all media was adjusted to 340 mOsm at pH7.4. All the media were stored at 4 °C, and were used within 2 weeks of production. Colonies were manually harvested at 60–80% confluence. Cells were then collected and dissociated into single cells using EDTA. Cells (1 × 10^6^ or 1 × 10^5^/well) were put onto 24- or 96-well ultra-low attachment plates (Corning, Inc., Corning, USA) to allow them to form aggregates in suspension in a CO_2_ incubator at 37 °C, in 5% CO_2_. Cell aggregates were cultured in E8 medium (Stem Cell Technologies, Vancouver, Canada) with daily medium change for 1–7 days.

### 4.6. Immunocytochemistry of iPSCs in Monolayer and Aggregate Culture

Control and *met*-IPSC were cultured on 8-well culture chambers were washed with PBS, fixed with 4% paraformaldehyde in PBS for 10-30 min, permeabilized with 0.2% Triton X-100 (Sigma, St. Louis, USA) in PBS and blocked in 10% serum. Primary antibodies were diluted in PBS 10% serum at the following concentrations: Brachyury (ab140661, 1:200, Abcam, Cambridge, UK), phospho-Met (3077S, 1:200, Ozyme, Saint-Cyr-l’École, France), PODXL (ab178566, 1:200, Abcam, Cambridge, UK), SIX2 (11562-1-AP, 1:200, Euromedex, Souffelweyersheim, France), and then washed in PBS. The samples were incubated with fluorescent secondary antibodies in antibody dilution buffer, then washed in PBS. Nuclei were labeled with DAPI (D9542, Sigma–Aldrich, St. Louis, USA) mounting medium. Visualization and capture were realized with a NIKON microscope (NIKON, Minato, Japan).

### 4.7. Immunohistochemistry of Mouse Kidney

Normal kidney in NSG mice was embedded in paraffin. Paraffin sections were deparaffinized and permeabilized with 0.2% Triton X-100 (Sigma, St. Louis, USA) in PBS and blocked in 10% serum. Primary antibodies were diluted in PBS containing 10% serum at the following concentrations: PODXL (ab178566, 1:200, Abcam, Cambridge, UK), VHL (SC-5575, 1:200, Bio-Techne, Minneapolis, USA) and washed three times in PBS. The sections were incubated with fluorescent secondary antibodies in antibody dilution buffer for 1 h, then washed three times in PBS. Nuclei were labeled with DAPI (D9542, Sigma–Aldrich, St. Louis, USA) mounting medium. Visualization and capture were realized with a NIKON microscope (NIKON, Minato, Japan).

### 4.8. Generation of Embryoid Bodies

Control or *met*-IPSC aggregates were plated on a Geltrex (STEMCELL Technologies, Inc., Vancouver, Canada) in 24 or 96-well plates or 8-well culture chambers. The aggregates were cultured in E8 medium (STEMCELL Technologies, Inc., Vancouver, Canada) with daily medium change for 6–7 days. Photographies were taken using a NIKON microscope (NIKON, Minato, Japan).

### 4.9. Whole-Mount Immunostaining of 3D Embryoid Bodies

Control or *met*-IPSC kidney embryoid bodies cultured on 96-well culture dishes or 8-well culture chambers were washed with PBS, fixed with 4% paraformaldehyde in PBS for 120 min, permeabilized with 0.2% Triton X-100 (Sigma, St. Louis, USA) in PBS and blocked in 10% serum. Primary antibodies were diluted in PBS 10% serum at the following concentrations: Nephrin (ab85379, 1:100, Abcam, Cambridge, UK), CD31 (ab24590, 1:50, Abcam, Cambridge, UK), PODXL (ab178566, 1:100, Abcam, Cambridge, UK), LTL (FL-132, 1:50, Clinisciences, Nanterre, France), VHL (SC-5575, 1:30, Bio-Techne, Minneapolis, USA), Phalloidin (A12381, 1:100, Invitrogen, Carlsbad, USA), cytokeratin 7 (ab9021, 1:100, Abcam, Cambridge, UK), TFE3 (ab179804, 1:100, Abcam, Cambridge, UK), Cubilin (ab191073, 1:50, Abcam, Cambridge, UK), phospho-Met (3077S, 1:100, Ozyme, Saint-Cyr-l’École, France), KDM4C (LS-C114684-100, 1:50, LSBio, Seattle, USA), BHLHE40 (ab70723, 1:50, Abcam, Cambridge, UK) and then washed in PBS. The samples were incubated with fluorescent secondary antibodies in antibody dilution buffer, then washed in PBS. Nuclei were labeled with DAPI (D9542, Sigma–Aldrich, St. Louis, USA) mounting medium. Visualization and capture were realized with a Leica confocal microscope type SP5-AOBS (Leica, Wetzlar, Germany) and NIKON microscope (NIKON, Minato, Japan). 

### 4.10. Immunohistochemistry of 3D Structures.

Kidney embryoid bodies generated in vitro were embedded in paraffin. Paraffin sections were deparaffinized and permeabilized with 0.2% Triton X-100 (Sigma, St. Louis, USA) in PBS and blocked in 10% serum. Primary antibodies were diluted in PBS containing 10% serum at the following concentrations: PODXL (ab178566, 1:200, Abcam), LTL (FL-1321, 1:200, Clinisciences, Nanterre, France), and washed three times in PBS. The sections were incubated with fluorescent secondary antibodies in antibody dilution buffer for 1 h, then washed three times in PBS. Nuclei were labeled with DAPI mounting medium. Visualization and capture were realized with a Leica confocal microscope (Leica, Wetzlar, Germany).

### 4.11. Transmission Electron Microscopy (TEM)

Kidney embryoid bodies were gently centrifuged and pelleted before the TEM process as follows. The cells were fixed in 2.5% glutaraldehyde in PBS for 1h at 4℃, washed in PBS, and fixed in 1% osmium tetroxide in PBS for 1h. They were dehydrated in ascending series of graded ethyl alcohols, then in acetone. Each sample was infiltrated with the resin before being embedded in epoxy resin and polymerized for 72 h. Semi-thin sections of about 0.5 to 1 µm were obtained and colored with Toluidine blue before being examined via a light microscope with an associated digital camera, hooked to a computer for image processing and editing (Leica DC 300). Ultra-thin sections of about 60/90 nm were contrasted with heavy metals (uranyl acetate and lead citrate) and were examined using a Jeol 1010 transmission electron microscope at an accelerated voltage of 80 kV. Images were photographed on digital images Gatan Digital Micrograph: brure Erlangen 500 w: camera and edited by Microsoft Power Point.

### 4.12. Functional Analysis of Proximal Tubules in Embryoid Bodies Containing Kidney Cells.

For dextran uptake assay, aggregates at day + 12 were cultured with 20 μg/mL of 10,000MW Dextran–Alexa 488-conjugated (D-22910, Thermo Fisher Scientific, Waltham, USA) for 48 h. Structures were then fixed in 4% PFA, washed three times in PBS. Nuclei were labeled with DAPI (D9542, Sigma–Aldrich, St. Louis, USA) mounting medium. Visualization and capture were realized with a NIKON microscope (NIKON, Minato, Japan).

### 4.13. Transcriptome Analyses of iPSC-Derived Embryoid Bodies

Microarray Clariom S human was done on total RNA from embryoid body samples (*met*-IPSC and control iPSC) in duplicates. Expression matrix was built with CEL files generated on Affymetrix Station and normalized by RMA method with Expression console software (Affymetrix, Santa Clara, USA) version 1.4.1.46 [35]. Gene set enrichment analysis was performed between conditions with MsigDb database version 6.0 [36].

### 4.14. Kidney Capsule Transplantation Experiments

Embryoid bodies containing kidney structures cultured for 12–14 days were transplanted beneath the renal capsule of 30–32-week-old male NSG mice (NOD.Cg-Prkdcscid Il2rgtm1Wjl/SzJ). 4 weeks after transplantation, mice were euthanized, kidneys were removed and processed for histology and immunofluorescence analysis.

### 4.15. Kidney Histology and Immunohistochemistry Analyses

After euthanasia, the kidneys containing the transplanted embryoid bodies were fixed in paraformaldehyde and embedded in paraffin. 4 μm sections were deparaffinized and then permeabilized with 0.2% Triton X-100 (Sigma, St. Louis, USA) in PBS and blocked 10% serum. Primary antibodies were diluted in PBS 10% serum at the following concentrations: Nephrin (ab85379, 1:200, Abcam, Cambridge, UK), CD31 (ab24590, 1:200, Abcam, Cambridge, UK), TFE3 (ab179804, 1:200, Abcam, Cambridge, UK), cytokeratin 7 (ab9021, 1:200, Abcam, Cambridge, UK), then washed three times in PBS. The sections were incubated with fluorescent secondary antibodies in antibody dilution buffer for 1 h, then washed three times in PBS. Nuclei were labeled with DAPI (D9542, Sigma–Aldrich, St. Louis, USA) mounting medium. Visualization and capture were realized with a NIKON microscope (NIKON, Minato, Japan).

### 4.16. Public Datasets

RNA-seq and genomic experiments from dataset of papillary renal cell carcinoma from TCGA consortium [12] were downloaded from Cbioportal database access center [37]. This analyzed dataset comprised 291 samples of PRCC tumors quantified by RNA-seq at level V2 Z-scores—21 patients were found mutated for c-*met* in this cohort (Table S5).

### 4.17. Transcriptome Analyses of iPSC Aggregates

Total RNA from *met*-IPSC (monolayer) and *met*-IPSC-A (aggregates) was extracted isolation RNA with the PureLink RNA Mini Kit (Thermo Fisher Scientific, Waltham, USA) by following manufacturer instructions. Quantification of RNA was performed with Nanodrop technology and RNA integrity was controlled with Agilent Bioanalyzer (Agilent technologies, Santa Clara, USA). Microarray probes were synthetized and labeled by linear amplification kit as Affymetrix manufacturer instructions to be hybridized to Human Clariom S microarray compatible with Affymetrix Station. Resulting scanned files (CEL files) were normalized with RMA method with Expression console version 1.4.6 (Affy*met*rix, Santa Clara, USA) [35].

### 4.18. Bioinformatics

Bioinformatics analysis was performed in R software environment version 3.0.2. Ranking product analysis was performed on transcriptome controlized matrix with implementation of one hundred of permutations [38] supervised by class description defined by culture conditions of the iPSC: *met*-IPSC (monolayer culture) versus *met*-IPSC-A. Expression heatmap was performed with made 4 bioconductor library by using Euclidean distances and Ward classification method [39]. Analysis of PRCC RNA-seq dataset was performed by machine learning with library pamr [40]. Protein-protein interaction network was built with Network Analyst application [41] on innateDB interaction database [42]. Functional inference on interaction network was enriched with KEGG database [43].

### 4.19. Immunohistochemistry Analysis of Primary Kidney Tumors

Two patients with c-*met*-mutated PRCC (Tumor Samples UPN4, UPN5) and primary PRCC tumors from three patients without c-*met* mutation (Tumor samples UPN1, UPN2 and UPN3) kidney tissue were embedded in paraffin. Paraffin sections were deparaffinized and then permeabilized with 0.2% Triton X-100 (Sigma, St. Louis, USA) in PBS and blocked 10% serum. Primary antibodies were diluted in PBS 10% serum at the following concentrations: KDM4C (LS-C114684-100, 1:200, LSBio, Seattle, USA), BHLHE40 (ab70723, 1:200, Abcam, Cambridge, UK) and then washed three times in PBS. The sections were incubated with fluorescent secondary antibodies in antibody dilution buffer for 1 h, then washed three times in PBS. Nuclei were labeled with DAPI (D9542, Sigma–Aldrich, St. Louis, USA) mounting medium. Visualization and capture were realized with a NIKON microscope (NIKON, Minato, Japan).

### 4.20. Toxicity Tests Using c-Met-Mutated iPSC-Derived Kidney Embryoid Bodies

Control or *met*-IPSC-derived kidney embryoid bodies cultured in 96-well plates were treated with cisplatin (500 g/mL) for 24h or with Sunitinib at low (20 g/mL) or high (500 g/mL) concentration for 96h. They were then fixed, and processed for immunofluorescence with KIM-1 antibodies (NBP1-7670, 1:100, Bio-Techne), PODXL (ab178566, 1:100, Abcam), LTL (FL-1321, 1:50, Clinisciences). Nuclei were labeled with DAPI (D9542, Sigma–Aldrich) mounting medium. Visualization and capture were realized with a Leica confocal microscope (Leica, Wetzlar, Germany).

## Supplementary Materials

Supplementary materials can be found at https://www.mdpi.com/1422-0067/20/19/4867/s1.

## References

[1] Cragun D., Pal T. (2013). Identification, Evaluation, and Treatment of Patients with Hereditary Cancer Risk within the United States. ISRN Oncol..

[2] Hadoux J., Féraud O., Griscelli F., Opolon P., Divers D., Gobbo E., Schlumberger M., Bennaceur-Griscelli A., Turhan A.G. (2016). Generation of an induced pluripotent stem cell line from a patient with hereditary multiple endocrine neoplasia 2A (MEN2A) syndrome with RET mutation. Stem Cell Res..

[3] Lee D.-F., Su J., Kim H.S., Chang B., Papatsenko D., Zhao R., Yuan Y., Gingold J., Xia W., Darr H. (2015). Modeling familial cancer with induced pluripotent stem cells. Cell.

[4] Griscelli F., Oudrhiri N., Feraud O., Divers D., Portier L., Turhan A.G., Bennaceur Griscelli A. (2017). Generation of induced pluripotent stem cell (iPSC) line from a patient with triple negative breast cancer with hereditary exon 17 deletion of BRCA1 gene. Stem Cell Res..

[5] Soyombo A.A., Wu Y., Kolski L., Rios J.J., Rakheja D., Chen A., Kehler J., Hampel H., Coughran A., Ross T.S. (2013). Analysis of Induced Pluripotent Stem Cells from a BRCA1 Mutant Family. Stem Cell Rep..

[6] Kim J., Hoffman J.P., Alpaugh R.K., Rhim A.D., Rhimm A.D., Reichert M., Stanger B.Z., Furth E.E., Sepulveda A.R., Yuan C.-X. (2013). An iPSC line from human pancreatic ductal adenocarcinoma undergoes early to invasive stages of pancreatic cancer progression. Cell Rep..

[7] Huang L., Holtzinger A., Jagan I., BeGora M., Lohse I., Ngai N., Nostro C., Wang R., Muthuswamy L.B., Crawford H.C. (2015). Ductal pancreatic cancer modeling and drug screening using human pluripotent stem cell- and patient-derived tumor organoids. Nat. Med..

[8] Chartier S., Méjean A., Richard S., Thiounn N., Vasiliu V., Verkarre V. (2017). Biphasic Squamoid Alveolar Renal Cell Carcinoma: 2 Cases in a Family Supporting a Continuous Spectrum With Papillary Type I Renal Cell Carcinoma. Am. J. Surg. Pathol..

[9] Self M., Lagutin O.V., Bowling B., Hendrix J., Cai Y., Dressler G.R., Oliver G. (2006). Six2 is required for suppression of nephrogenesis and progenitor renewal in the developing kidney. EMBO J..

[10] Kobayashi A., Valerius M.T., Mugford J.W., Carroll T.J., Self M., Oliver G., McMahon A.P. (2008). Six2 defines and regulates a multipotent self-renewing nephron progenitor population throughout mammalian kidney development. Cell Stem Cell.

[11] Delahunt B., Eble J.N. (1997). Papillary renal cell carcinoma: a clinicopathologic and immunohistochemical study of 105 tumors. Mod. Pathol. Off. J. U. S. Can. Acad. Pathol. Inc.

[12] Linehan W.M., Spellman P.T., Ricketts C.J., Creighton C.J., Fei S.S., Davis C., Wheeler D.A., Murray B.A., Schmidt L., Cancer Genome Atlas Research Network (2016). Comprehensive Molecular Characterization of Papillary Renal-Cell Carcinoma. N. Engl. J. Med..

[13] Lubensky I.A., Schmidt L., Zhuang Z., Weirich G., Pack S., Zambrano N., Walther M.M., Choyke P., Linehan W.M., Zbar B. (1999). Hereditary and Sporadic Papillary Renal Carcinomas with c-met Mutations Share a Distinct Morphological Phenotype. Am. J. Pathol..

[14] Siegel R.L., Miller K.D., Jemal A. (2016). Cancer statistics, 2016. CA. Cancer J. Clin..

[15] Lam A.Q., Freedman B.S., Morizane R., Lerou P.H., Valerius M.T., Bonventre J.V. (2014). Rapid and efficient differentiation of human pluripotent stem cells into intermediate mesoderm that forms tubules expressing kidney proximal tubular markers. J. Am. Soc. Nephrol. JASN.

[16] Takasato M., Er P.X., Chiu H.S., Maier B., Baillie G.J., Ferguson C., Parton R.G., Wolvetang E.J., Roost M.S., de Sousa Lopes S.M.C. (2015). Kidney organoids from human iPS cells contain multiple lineages and model human nephrogenesis. Nature.

[17] Morizane R., Lam A.Q., Freedman B.S., Kishi S., Valerius M.T., Bonventre J.V. (2015). Nephron organoids derived from human pluripotent stem cells model kidney development and injury. Nat. Biotechnol..

[18] Dutta D., Heo I., Clevers H. (2017). Disease Modeling in Stem Cell-Derived 3D Organoid Systems. Trends Mol. Med..

[19] Davidowitz E.J., Schoenfeld A.R., Burk R.D. (2001). VHL Induces Renal Cell Differentiation and Growth Arrest through Integration of Cell-Cell and Cell-Extracellular Matrix Signaling. Mol. Cell. Biol..

[20] Luckett W.P. (1975). The development of primordial and definitive amniotic cavities in early rhesus monkey and human embryos. Am. J. Anat..

[21] Takabatake Y., Sugiyama T., Kohara H., Matsusaka T., Kurihara H., Koni P.A., Nagasawa Y., Hamano T., Matsui I., Kawada N. (2009). The CXCL12 (SDF-1)/CXCR4 axis is essential for the development of renal vasculature. J. Am. Soc. Nephrol. JASN.

[22] Yu W., Zhang W., Jiang Y., Wang Y., Li Y., Wang J., Sun L., Ran W., Li H. (2013). Clinicopathological, genetic, ultrastructural characterizations and prognostic factors of papillary renal cell carcinoma: New diagnostic and prognostic information. Acta Histochem..

[23] Tsuda M., Davis I.J., Argani P., Shukla N., McGill G.G., Nagai M., Saito T., Laé M., Fisher D.E., Ladanyi M. (2007). TFE3 Fusions Activate MET Signaling by Transcriptional Up-regulation, Defining Another Class of Tumors as Candidates for Therapeutic MET Inhibition. Cancer Res..

[24] Chevarie-Davis M., Riazalhosseini Y., Arseneault M., Aprikian A., Kassouf W., Tanguay S., Latour M., Brimo F. (2014). The morphologic and immunohistochemical spectrum of papillary renal cell carcinoma: study including 132 cases with pure type 1 and type 2 morphology as well as tumors with overlapping features. Am. J. Surg. Pathol..

[25] Garcia J., Lizcano F. (2016). KDM4C Activity Modulates Cell Proliferation and Chromosome Segregation in Triple-Negative Breast Cancer. Breast Cancer Basic Clin. Res..

[26] Krill-Burger J.M., Lyons M.A., Kelly L.A., Sciulli C.M., Petrosko P., Chandran U.R., Kubal M.D., Bastacky S.I., Parwani A.V., Dhir R. (2012). Renal Cell Neoplasms Contain Shared Tumor Type–Specific Copy Number Variations. Am. J. Pathol..

[27] Han W.K., Bailly V., Abichandani R., Thadhani R., Bonventre J.V. (2002). Kidney Injury Molecule-1 (KIM-1): A novel biomarker for human renal proximal tubule injury. Kidney Int..

[28] Zarrabi K., Paroya A., Wu S. (2019). Emerging therapeutic agents for genitourinary cancers. J. Hematol. Oncol.J Hematol Oncol.

[29] Wang Q., Yang S., Wang K., Sun S.-Y. (2019). MET inhibitors for targeted therapy of EGFR TKI-resistant lung cancer. J. Hematol. Oncol..

[30] Berry W.L., Janknecht R. (2013). KDM4/JMJD2 histone demethylases: epigenetic regulators in cancer cells. Cancer Res..

[31] Cheung N., Fung T.K., Zeisig B.B., Holmes K., Rane J.K., Mowen K.A., Finn M.G., Lenhard B., Chan L.C., So C.W.E. (2016). Targeting Aberrant Epigenetic Networks Mediated by PRMT1 and KDM4C in Acute Myeloid Leukemia. Cancer Cell.

[32] Sato F., Bhawal U.K., Yoshimura T., Muragaki Y. (2016). DEC1 and DEC2 Crosstalk between Circadian Rhythm and Tumor Progression. J. Cancer.

[33] Ivanova A., Liao S.-Y., Lerman M.I., Ivanov S., Stanbridge E.J. (2005). STRA13 expression and subcellular localisation in normal and tumour tissues: implications for use as a diagnostic and differentiation marker. J. Med. Genet..

[34] Telliam G., Féraud O., Griscelli F., Opolon P., Divers D., Bennaceur-Griscelli A., Turhan A.G. (2016). Generation of an induced pluripotent stem cell line from a patient with chronic myeloid leukemia (CML) resistant to targeted therapies. Stem Cell Res..

[35] Irizarry R.A., Bolstad B.M., Collin F., Cope L.M., Hobbs B., Speed T.P. (2003). Summaries of Affymetrix GeneChip probe level data. Nucleic Acids Res..

[36] Subramanian A., Tamayo P., Mootha V.K., Mukherjee S., Ebert B.L., Gillette M.A., Paulovich A., Pomeroy S.L., Golub T.R., Lander E.S. (2005). Gene set enrichment analysis: a knowledge-based approach for interpreting genome-wide expression profiles. Proc. Natl. Acad. Sci. USA.

[37] Gao J., Aksoy B.A., Dogrusoz U., Dresdner G., Gross B., Sumer S.O., Sun Y., Jacobsen A., Sinha R., Larsson E. (2013). Integrative analysis of complex cancer genomics and clinical profiles using the cBioPortal. Sci. Signal..

[38] Breitling R., Armengaud P., Amtmann A., Herzyk P. (2004). Rank products: a simple, yet powerful, new method to detect differentially regulated genes in replicated microarray experiments. FEBS Lett..

[39] Culhane A.C., Thioulouse J., Perrière G., Higgins D.G. (2005). MADE4: an R package for multivariate analysis of gene expression data. Bioinforma. Oxf. Engl..

[40] Tibshirani R., Hastie T., Narasimhan B., Chu G. (2002). Diagnosis of multiple cancer types by shrunken centroids of gene expression. Proc. Natl. Acad. Sci. USA.

[41] Xia J., Gill E.E., Hancock R.E.W. (2015). NetworkAnalyst for statistical, visual and network-based meta-analysis of gene expression data. Nat. Protoc..

[42] Breuer K., Foroushani A.K., Laird M.R., Chen C., Sribnaia A., Lo R., Winsor G.L., Hancock R.E.W., Brinkman F.S.L., Lynn D.J. (2013). InnateDB: systems biology of innate immunity and beyond—Recent updates and continuing curation. Nucleic Acids Res..

[43] Ogata H., Goto S., Sato K., Fujibuchi W., Bono H., Kanehisa M. (1999). KEGG: Kyoto Encyclopedia of Genes and Genomes. Nucleic Acids Res..

