# Supplementary material for: iPSC-Derived Embryoid Bodies as Models of c-Met-Mutated Hereditary Papillary Renal Cell Carcinoma
Source: Int J Mol Sci. 2019 Sep 30;20(19):4867. doi: 10.3390/ijms20194867 (PMC6801716; doi:10.3390/ijms20194867)
Supplement: Supplementary file 1 [file ijms-20-04867-s001.zip › ijms-597613-FINAL-supplementary/Hwang et al Suppl Tables 1 to 5/Hwang et al Table S1.docx]

**Supplementary Table 4 The characteristics of the papillary renal cell carcinoma patients in the study.**

| **Patients** | **Birth**  **(year)** | **Gender**  **(Male/female)** | **c-*met*-mutated PRCC** |
| --- | --- | --- | --- |
| **UPN1** | **1970** | **Male** | **-** |
| **UPN2** | **1969** | **Male** | **-** |
| **UPN3** | **1952** | **Male** | **-** |
|  |  |  |  |
| **UPN4** | **-** | **-** | c.A3523G, p.His1112Arg |
| **UPN5** | **1933** | **Female** | c.3900 G>A, p.Val1238Ile |
|  |  |  |  |
