# Supplementary material for: iPSC-Derived Embryoid Bodies as Models of c-Met-Mutated Hereditary Papillary Renal Cell Carcinoma
Source: Int J Mol Sci. 2019 Sep 30;20(19):4867. doi: 10.3390/ijms20194867 (PMC6801716; doi:10.3390/ijms20194867)
Supplement: Supplementary file 1 [file ijms-20-04867-s001.zip › ijms-597613-FINAL-supplementary/ijms-597613-supplementary-figures.docx]

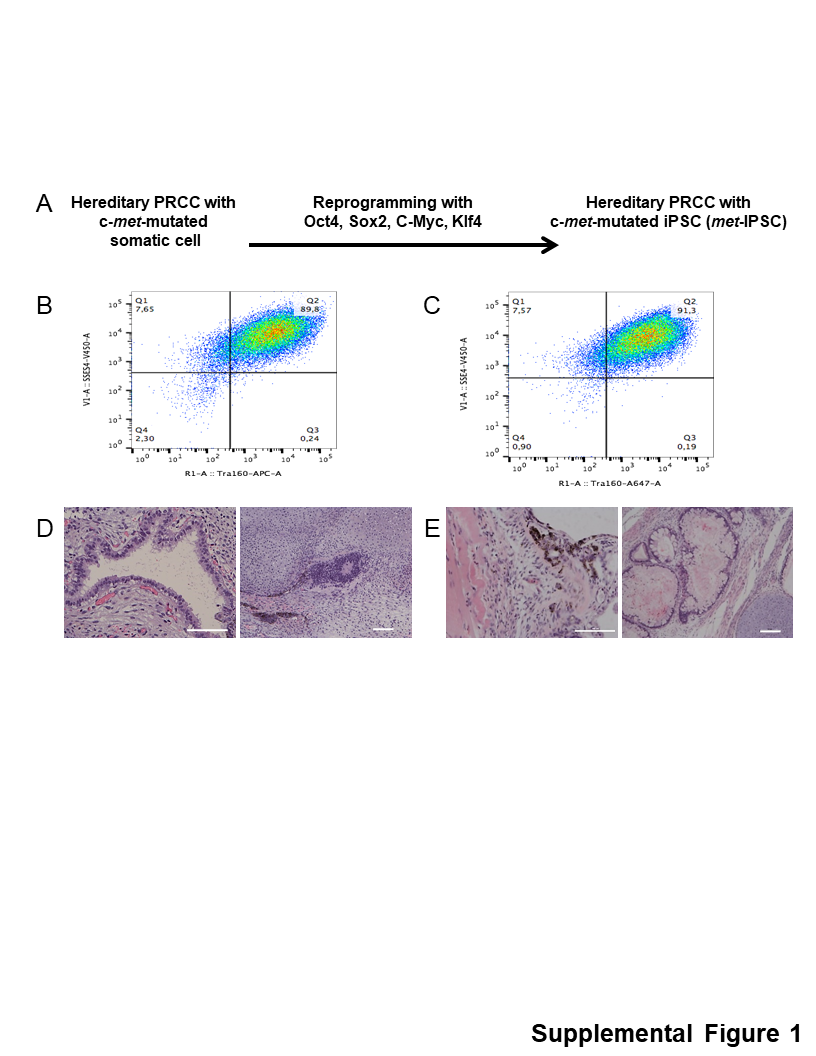


**Supplemental Figure 1.** Generation and characterization of *met*-IPSC using immunophenotyping and teratoma assays, related to Figure 1.(A) Protocol used for the generation of *met*-IPSC using Sendaï-virus mediated pluripotent gene transfer. (B-C) FACS analysis of control (left panel) or *met*-IPSC (right panel) using pluripotency markers Tra-1-60 and SSEA4. (D-E) H&E staining section of control (left panel) or *met*-IPSC (right panel) showing derivatives of all three germ layers. Scale bar : 100 µm.


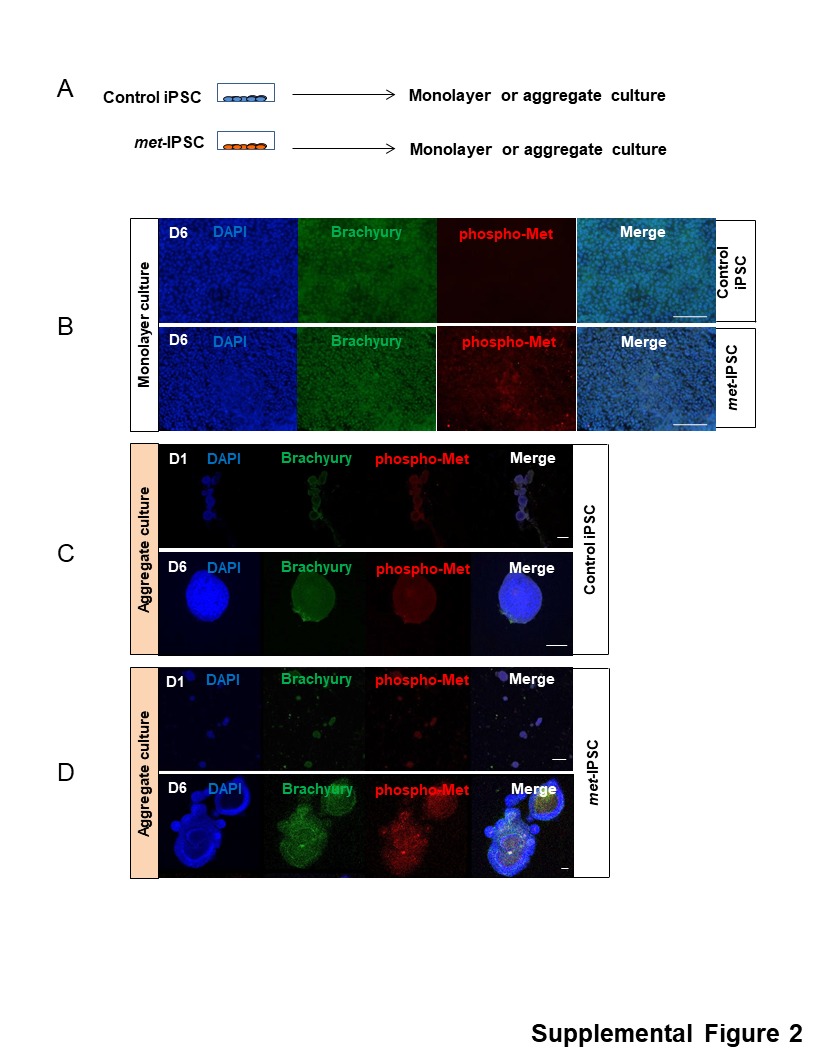


**Supplemental Figure 2.** Immunocytochemistry analyses of *met*-IPSC aggregates and evaluation of phospho-Met expression, related to Figure 1. (A) Schematic representation of control and *met*-IPSC grown in monolayer cultures with and without low attachment conditions generating aggregates. (B). Immunocytochemistry for Brachyury, phospho-Met and DAPI on day 6 of control iPSC or *met*-IPSC monolayer culture. A faint phospho-MET expression is detected in *met*-IPSC aggregates. (C-D) Immunocytochemistry analyses for Brachyury and phospho-Met expression on day 1 and 6 of control (C) or *met*-IPSC aggregates revealing similar levels of phospho-Met expression in both conditions. Scale bar : 100 μm.


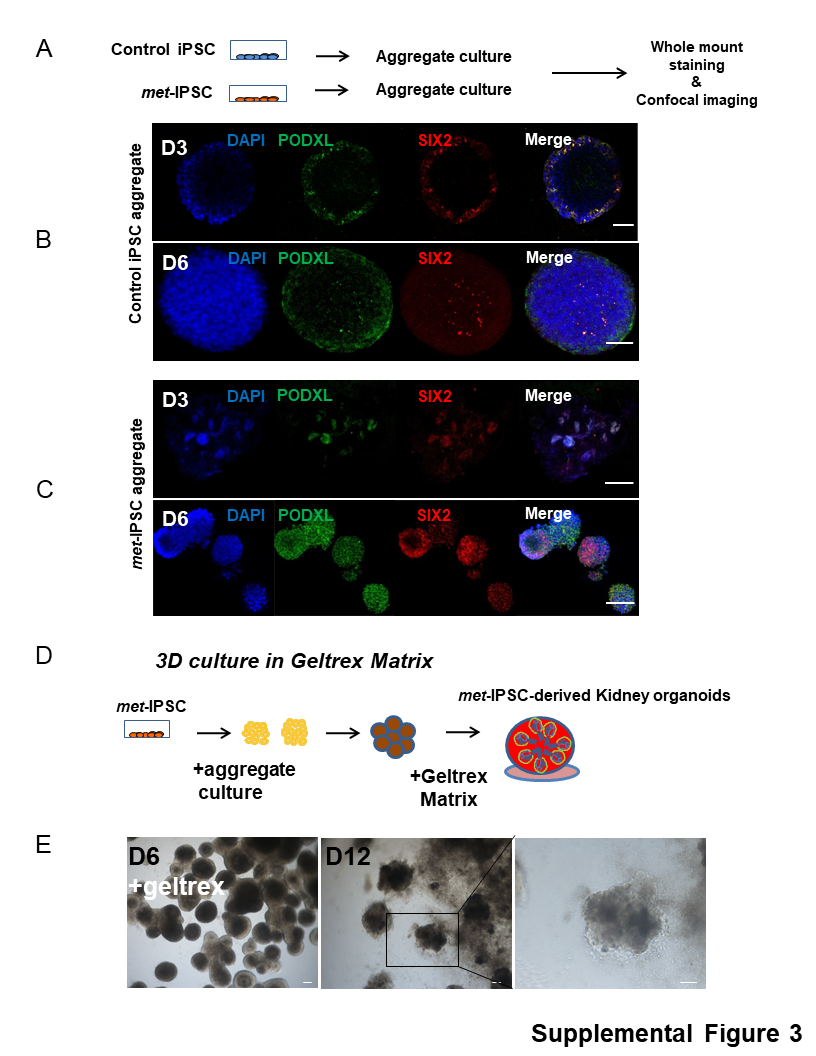


**Supplemental Figure 3.** Appearance of kidney differentiation markers in aggregate cultures to optimize kidney embryoid bodies generation in 3D cultures, related to Figure 1. (A) Schematic representation of the experiments designed to generate control iPSC and *met*-IPSC in aggregate cultures performed in low attachment plates. (B-C) Whole-mount immunostaining for PODXL, SIX2 and DAPI of control iPSC aggregates on day 3 and day 6 showing the appearance of kidney markets at day+3, with increased expression at day+6 in both control and *met*-IPSC. Scale bar : 50 μm. (D) Schema of the protocol used for of 3D cultures in Geltrex, based on the results obtained in aggregate cultures with optimal kidney differentiation at day+6. (E) 3D cultures at day+6 in the presence of Geltrex allowed the generation structures in which kidney differentiation markers were found (See Figures 4) Scale bar : 100 μm.


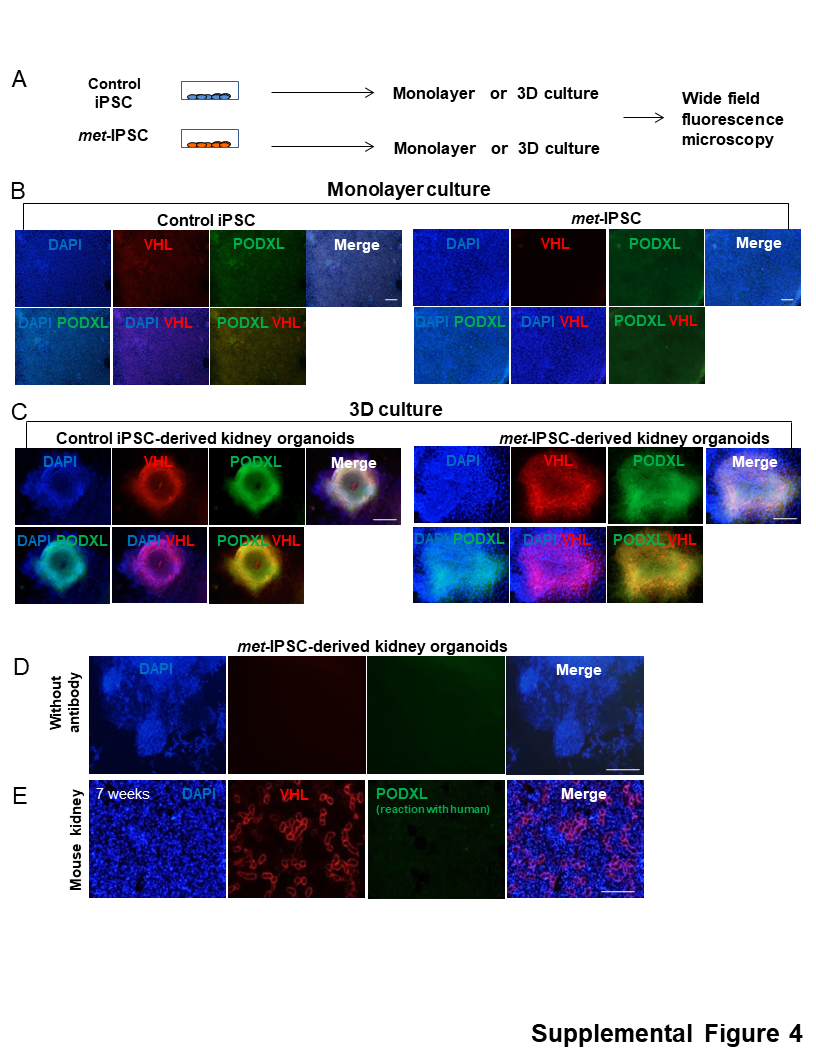


**Supplemental Figure 4.** Self-organizing kidney embryoid bodies are detected only in 3D cultures, related to Figure 1. (A) Schematic representation of experiments designed performed in control iPSC and *met*-IPSC monolayer or 3D culture. (B) Immunocytochemistry for PODXL, VHL and DAPI of iPSC in monolayer culture on day 6. The use of PODXL antibodies does not allow the detection of kidney differentiation. (C) Whole-mount immunostaining of embryoid bodies in 3D cultures followed by staining with PODXL, VHL allows detection of kidney differentiation markets at day+12. (D) Control experiment showing whole-mount immunostaining without 1^st^ antibody of *met*-IPSC-derived kidney embryoid bodies 3D culture on day 12. (E) Immunohistochemistry for PODXL, VHL and DAPI of mouse kidney. Scale bar : 100 μm.


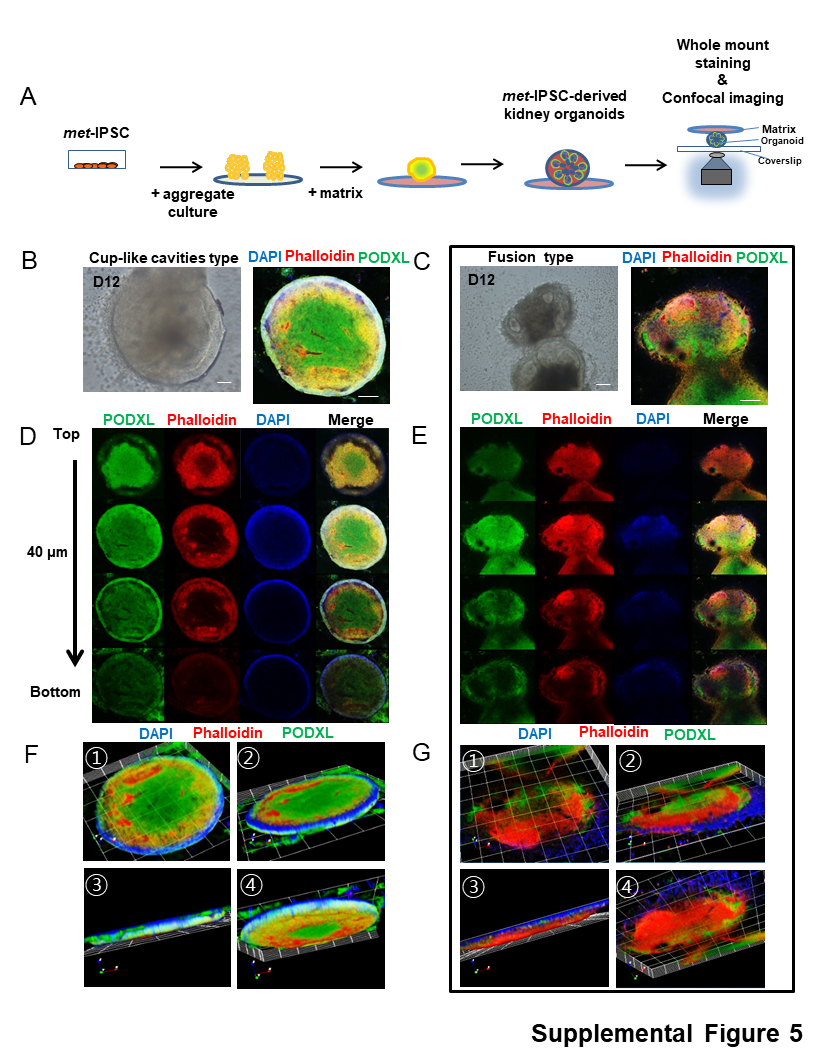


**Supplemental Figure 5. Self-organizing iPSC-derived kidney embryoid bodies, related to Figure 1.** (A) Schematic representation of the experiments designed to generate *met*-IPSC in aggregate culture. (B-C) Optical image of *met*-IPSC-derived kidney embryoid bodies and confocal image of whole-mount immunostaining for PODXL, phalloidin and DAPI. Scale bar : 100 μm. (D-E) Confocal image of cup-like cavities and fusion type. (F-G) 3D rotation image of cup-like cavities and fusion type.


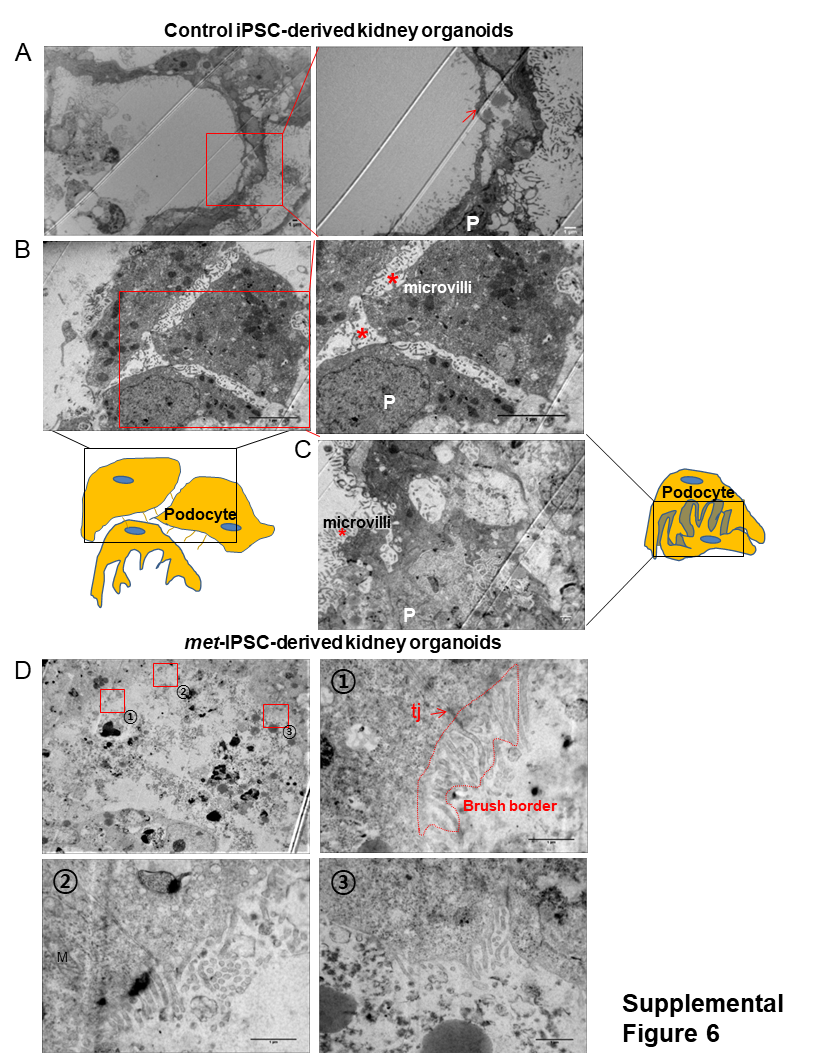


**Supplemental Figure 6.** Electron microscopy of iPSC-derived kidney embryoid bodies, related to Figure 3. (A-C) Representative electron microscopy images glomerulus region of control iPSC-derived kidney embryoid bodies, podocyte-like cells (P), glomerular basement membrane (arrow), microvilli (*). (D) Representative electron microscopy images tubule region of *met*-IPSC-derived kidney embryoid bodies, tight junctions (tj), mitochondria (M). Scale bar : 1 μm.


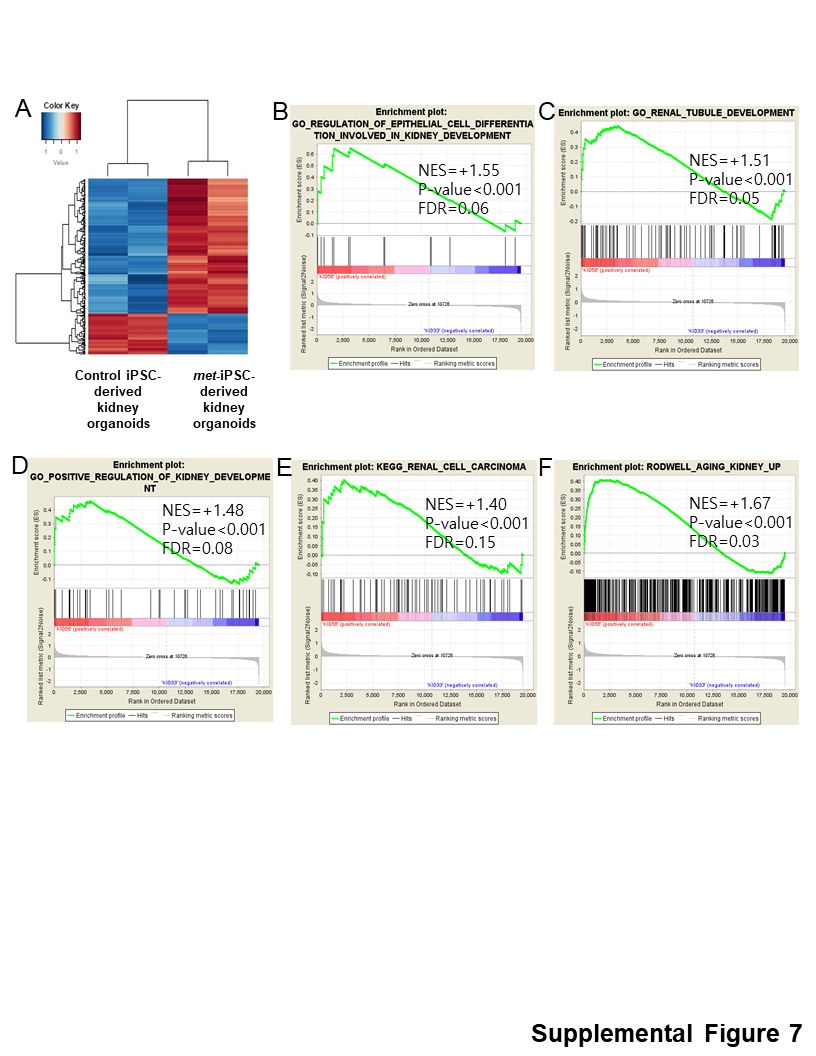


**Supplemental Figure 7.F**unctional enrichment of differentially expressed genes between control versus *met*-IPSC-derived kidney embryoid bodies, related to Figure 4. (A-D) Kidney development gene sets enriched in *met*-IPSC-derived kidney embryoid bodies versus control iPSC-derived kidney embryoid bodies, NES: normalized enriched score, FDR: False Discovery Rate. (E and F) Gene sets enriched in *met*-IPSC- derived kidney embryoid bodies. versus control iPSC-derived kidney embryoid bodies, NES: normalized enriched score, FDR: False Discovery Rate.
